# Data integration reveals dynamic and systematic patterns of breeding habitat use by a threatened shorebird

**DOI:** 10.1038/s41598-023-32886-w

**Published:** 2023-04-13

**Authors:** Kristen S. Ellis, Michael J. Anteau, Garrett J. MacDonald, Rose J. Swift, Megan M. Ring, Dustin L. Toy, Mark H. Sherfy, Max Post van der Burg

**Affiliations:** grid.2865.9 0000000121546924 U.S. Geological Survey, Northern Prairie Wildlife Research Center, 8711 37th St SE, Jamestown, ND 58401 USA

**Keywords:** Biogeography, Conservation biology, Ecological modelling, Wetlands ecology

## Abstract

Incorporating species distributions into conservation planning has traditionally involved long-term representations of habitat use where temporal variation is averaged to reveal habitats that are most suitable across time. Advances in remote sensing and analytical tools have allowed for the integration of dynamic processes into species distribution modeling. Our objective was to develop a spatiotemporal model of breeding habitat use for a federally threatened shorebird (piping plover, *Charadrius melodus*). Piping plovers are an ideal candidate species for dynamic habitat models because they depend on habitat created and maintained by variable hydrological processes and disturbance. We integrated a 20-year (2000–2019) nesting dataset with volunteer-collected sightings (eBird) using point process modeling. Our analysis incorporated spatiotemporal autocorrelation, differential observation processes within data streams, and dynamic environmental covariates. We evaluated the transferability of this model in space and time and the contribution of the eBird dataset. eBird data provided more complete spatial coverage in our study system than nest monitoring data. Patterns of observed breeding density depended on both dynamic (e.g., surface water levels) and long-term (e.g., proximity to permanent wetland basins) environmental processes. Our study provides a framework for quantifying dynamic spatiotemporal patterns of breeding density. This assessment can be iteratively updated with additional data to improve conservation and management efforts, because reducing temporal variability to average patterns of use may cause a loss in precision for such actions.

## Introduction

Evaluating spatial and temporal patterns in species occurrences has been a challenging but necessary endeavor in macroecology and conservation biology, particularly for rare or declining species that rely on dynamic or patchily-distributed habitats. Approximating species’ distributions or densities often involves relating presence-only, detection/non-detection, or count data to biotic and abiotic characteristics of the environment^1^. These predictions can be valuable for assessing habitat suitability or informing the conservation of rare species at locations where occupancy information is uncertain^2^. One approach commonly used in conservation planning is to produce static realizations of species’ distributions by pooling spatial data across time, because there may not be enough occurrence data to build models of species’ responses to fluctuating conditions^3^. However, ecological processes underlying species’ distributions are complex, and averaging temporal variation in habitat suitability or occurrences of highly mobile species may lead to a loss in precision needed for adaptive conservation planning^2,4^.

Linkages between environmental factors and species’ distributions or densities may vary over time for many reasons, including climate shifts, changes in population dynamics (e.g., rates of dispersal), or community interactions^5–8^. For example, phases of substantial dry or wet weather conditions can induce variable occurrence patterns across a diversity of avian species^9,10^. Further, spatial covariation caused by the geographic proximity of occurrences can vary as organisms move^11^. If unaccounted for, such unobserved processes can bias estimates of habitat associations, consequently decreasing the predictive ability of species distribution models^12,13^. The practical utility of a species distribution model for resource managers and conservation practitioners therefore largely depends on prediction accuracy^14^, which can influence land-use planning^15^ and the management of populations^16^.

Often, the scope of interest for understanding patterns in species occurrences extends beyond the temporal or spatial limits of an established survey or monitoring program^1^. The transferability of predictions into new locations or time periods can depend on a balance between over-parameterized models with high predictive accuracy and models with limited complexity^17,18^. Transferability can further depend on species’ life histories, spatial extent of distributions, habitat heterogeneity, and degree of extrapolation^19^. The development of procedures that integrate multiple sources of data into species distribution models has become increasingly more common, because this allows researchers to improve parameter estimates, generalize patterns across monitoring schemes, and extend the scope of inference^1,20–24^. A common theme within data integration research involves the use of openly available, opportunistic data collected by members of the public^23^. Opportunistic data are typically plentiful and can resolve spatial gaps where structured surveys do not occur, yet generally suffer from detection biases^25,26^. Therefore, methods accounting for differential observation processes while estimating a latent species distribution provide opportunities to incorporate data with diverse strengths and weaknesses^21^.

We applied an integrated point process model^21^ to evaluate annual dynamics in the breeding habitat use and density of an uncommon migratory bird of high conservation interest across a broad region. Point process models are used to describe point locations in continuous space and estimate an intensity surface of the density of observations (or patterns of relative abundance) within an area^27^. Moreover, point process models simplify potential issues around scale dependence when integrating data from multiple sources, as the measured spatial accuracy of points can be retained, eliminating the need to discretize the study area into spatial units^21^. Using both nesting locations and opportunistic observations (eBird)^26^, we modeled piping plover (*Charadrius melodus*) breeding densities in the United States Prairie Pothole Region (PPR) across a 20-year period. Piping plovers (hereafter ‘plovers’) are a federally (USA) listed migratory shorebird and a portion of the species breeds on unvegetated shorelines of PPR wetlands. The availability of these breeding habitats is sensitive to changes in precipitation and temperature, and the persistence of unvegetated wetland shorelines in the PPR is uncertain due to interacting effects of changes in land use and climate^28^. Therefore, this study system offers an opportunity to assess the degree of spatiotemporal variability in breeding habitat use and density in response to the dynamic availability of habitats. Further, we developed procedures for continued monitoring of plover habitats in the PPR, allowing for more dynamic approaches to conservation when and where it is needed most.

## Methods

### Study area

**Figure 1:**
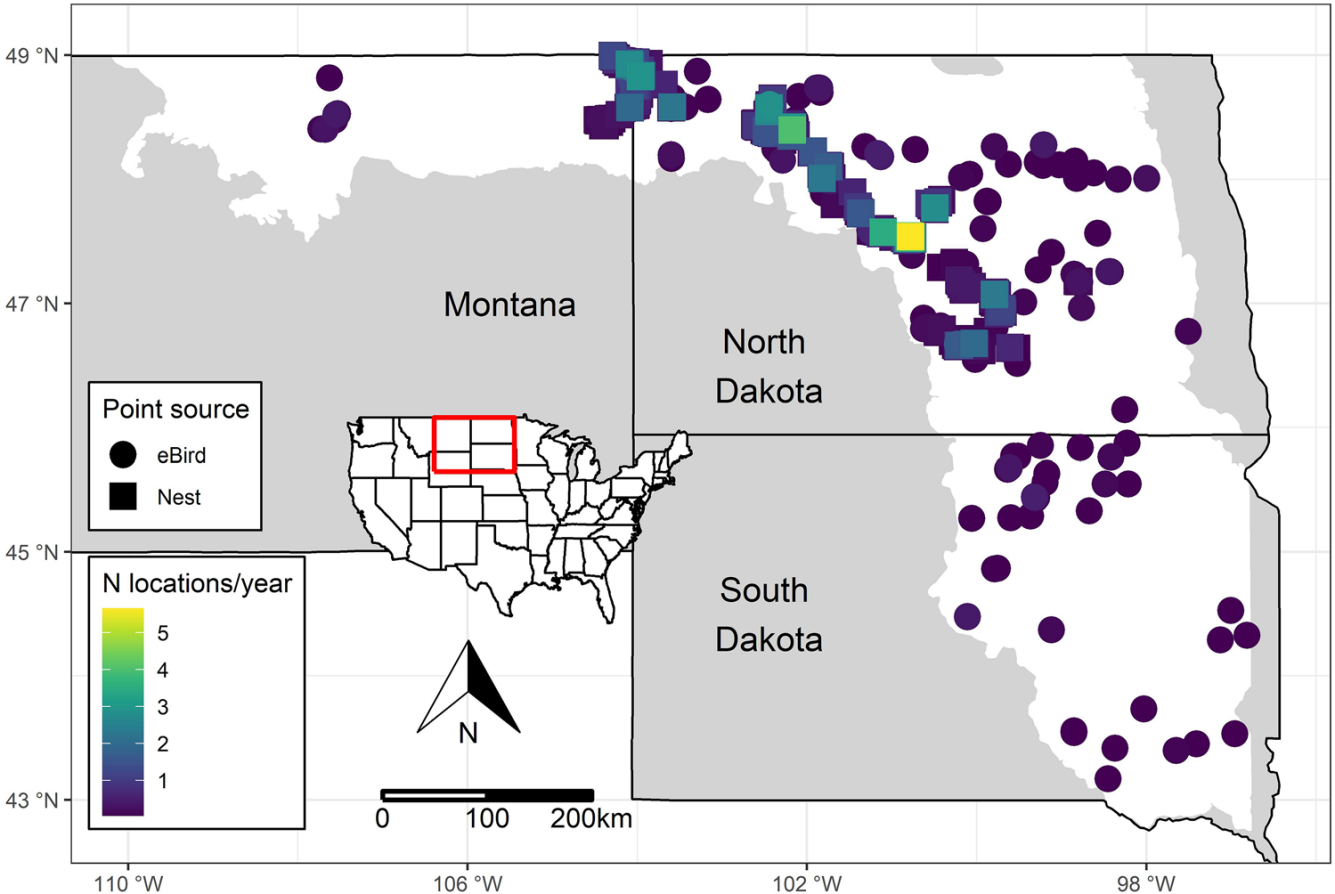
Study area in the north central United States including North Dakota, South Dakota, and eastern Montana. The white polygon indicates the extent of the Prairie Pothole Region included in our study. Points indicate the spatial distribution of data used from piping plover (*Charadrius melodus*) nest locations and eBird observations collected between 2000 and 2019. Each point represents a 300 m circular area and the color indicates the average number of data locations per year in each area across our study period. Maps were generated using R (version 4.1.3)^55^.

The PPR covers more than 700,000 km^2^ of the North American midcontinent. This region was historically characterized by millions of small, depressional wetlands and perennial grasslands, although wetland drainage and conversion of grasslands to agricultural fields has altered much of this ecosystem^29,30^. The abundance and diversity of wetlands in the PPR provide critical breeding and stopover habitats for a myriad of waterfowl, shorebird, and other wetland-dependent species^31–33^. Our study focused on private and public lands in the PPR in eastern Montana, North Dakota, and South Dakota (Fig. 1). This area included the Aspen Parkland/Northern Glaciated Plains and Northwestern Glaciated Plains North American Level III ecoregions. We excluded regions that are often considered to be within the PPR including Minnesota, Iowa, the Lake Agassiz Plain ecoregion of North Dakota, and western Montana from our study extent because observations of plovers in these areas across our study period were nonexistent or too infrequent to model.

### Nesting data

We collected piping plover nest locations from surveys conducted by the U.S. Fish and Wildlife Service (USFWS) and the U.S. Geological Survey (USGS) between 2000 and 2019. Nest surveys were conducted at approximately 180 wetlands within the Alkali Lakes Core Area^34^, as these wetlands had relatively consistent monitoring efforts across our study period (Fig. S1). Between late April and early August, USFWS (2000–2019) and joint USFWS—USGS (2014–2019) crews searched shoreline habitats within the Alkali Lakes Core Area and used behavioral cues of adults to locate nest sites. We defined a nest site as a scrape with at least one egg being incubated by an adult. Once located, the coordinates of individual nest sites were recorded and used as point locations in our analysis.

### eBird data

eBird is a publicly-available online repository of bird observations voluntarily reported by members of the community^26^. Observers submit records as checklists with counts of species that were seen. We used checklist metadata to obtain spatial coordinates, survey dates, and information about the sampling effort (checklist duration, distance traveled, and number of observers). We retrieved eBird data on 1 February 2022 and restricted data to the breeding season (May–July) for the period 2000–2021. We filtered eBird data according to best practices^35^. These practices included only considering checklists where the distance traveled was < 5 km, surveys were conducted between 5:00 a.m. and 9:00 p.m., were collected over a period of less than 5 h, and with no more than 10 observers. We included incomplete checklists where plovers were observed because the data were treated as presence-only locations (see modeling details below). For our analysis, eBird locations represented sightings of adults that were presumed breeders, and not actual nest locations. We assumed that eBird sightings of plovers indicated suitable breeding habitats because previous work in the Great Plains has shown that breeding and nonbreeding plovers are routinely observed during breeding season surveys^36,37^. Therefore, habitats selected by nonbreeding plovers during the breeding season are likely similar to those used for nesting. However, during the breeding season, nonbreeding plovers on the Atlantic coast use beach habitats at a fine-scale differently than breeding plovers^38^.

### Remotely sensed explanatory layers

We tested the explanatory strength of a suite of habitat characteristics that were considered a priori to be associated with plover breeding intensity (Table 1; Fig. S2). Plover nest and eBird locations were primarily located near wetlands that were classified as lakes according to the USFWS’s National Wetlands Inventory. The mean distance to lake edges was 102 m for nests (s.d. = 311 m; range = 0–4288 m) and 1,770 m for eBird locations (s.d. = 3,844 m; range = 0–18,513 m). Reduced spatial accuracy of eBird observations (e.g., routes varied in distance traveled) relative to nest locations likely contributed to their difference in mean distances to lake edges. We created a distance-based raster where each 30 m pixel indicated the distance from the center of the pixel to the nearest lake edge, and we included this layer as a covariate (Table 1). We anticipated that the effect of lakes would decline at increasing distances, and additionally calculated the distance to lakes using an exponential function, $1-\mathrm{exp}(-d/\mu )$, where *d* was the distance, and *μ* was the mean distance value from all eBird and nest points^39^.

**Table 1:** Candidate covariates used to develop piping plover breeding density models and their data sources.

Category	Source	Temporal resolution	Candidate covariates
Site accessibility	Gravel and paved roads^50^	Static	Road density within 5 km
Checklist effort	eBird^26^	Annual	Checklist duration; Checklist distance
Anthropogenic	Gravel and paved roads^50^	Static	Distance to roads (m); Binary classification: > 100 m from roads = 1, < 100 m from roads = 0
Anthropogenic	Settlements^50^	Static	Distance to human settlements (m); Binary classification: > 1 km from settlements = 1, < 1 km from settlements = 0
Topography	DEM (U.S. Geological Survey National Map)	Static	Slope^a^
Hydrology	DSWE^43^	Annual	Percent surface water^a,b^
Hydrology	NWI	Static	Distance to permanent lake features (m); Distance to permanent lake features—exponential function
Vegetation	NDVI (Landsat)	Annual	NDVI^a,b^
Land cover	NLCD^49^	Every 2–3 years	Percent crop and hay pasture^a^; Distance to trees (m)

Site accessibility and checklist effort covariates were only applied to the eBird observation process and remaining covariates were applied to the latent species distribution process. Temporal resolution refers to the frequency at which data layers were produced across our study period. *DEM* Digital elevation model; *DSWE* Dynamic surface water extent; *NWI* National wetlands inventory; *NDVI* Normalized difference vegetation index; *NLCD* National land cover database.

^a^These covariates were considered at multiple spatial extents: 30 m, all adjacent pixels, 90 m, 150 m, 300 m, and 750 m.

^b^These covariates included a candidate quadratic effect.

Breeding activities of plovers primarily occur along unvegetated shorelines^40–42^, and we expected that accurately classifying surface water and vegetation coverage would be crucial in predicting breeding habitats. Developing dynamic predictions for each year of our study required annual data layers describing water-level conditions and vegetation coverage. Because data collection for this project began in 2000, we obtained annual water and vegetation coverage layers using successive Landsat mosaics within the breeding season, as the Landsat record facilitated a continuous source of data across our study period (Landsat 5: 2000–2011, Landsat 7: 2012–2013, Landsat 8: 2014–2021). To describe annual water-level conditions, we used the Dynamic Surface Water Extent (DSWE) product^43^ from Landsat imagery (acquisition dates: 1 April–30 September). The DSWE algorithm produces a raster layer where pixel values can be integers ranging between 0 and 4 representing varying levels of confidence in the presence of water (0 = not water, 1 = water–high confidence, 2 = water–moderate confidence, 3 = partial surface water–conservative, 4 = partial surface water–aggressive). We reclassified DSWE layers to only include high confidence water (pixels with a value of 1 at any point during the imagery acquisition window remained 1 and all other pixels were reclassified to 0). To identify unvegetated or sparsely vegetated patches, we used the Normalized Difference Vegetation Index (NDVI) as a representation of vegetation coverage^40^, using the maximum NDVI value from Landsat mosaics across all cloud-free observations of Landsat for the breeding season (acquisition dates: 1 May–31 August).

We expected that plover breeding activities would occur on substrates with a flat slope^40,44,45^. We applied the slope tool in Google Earth Engine^46^ to measure the degree of inclination of a 30 m resolution digital elevation model obtained from the U.S. Geological Survey National Elevation Dataset (http://viewer.nationalmap.gov/viewer/). We hypothesized that land cover characteristics surrounding wetlands could influence plover breeding habitat use, potentially due to agricultural land use changes influencing wetland dynamics^47^ or avian nest predators using trees for perching near wetlands (although effects of trees may be more relevant to reproductive success than habitat use)^48^. We used the National Land Cover Database (NLCD) layers, which were generated from 2001 to 2019 at 2–3-year intervals^49^. We summarized NLCD layers into crop and pasture layers (if a raster cell was categorized as pasture/hay or cultivated crops, it received a value of 1, all others were 0) and the Euclidean distance to trees (deciduous forest, evergreen forest, or mixed forest). Lastly, we hypothesized that plovers would avoid wetlands near human settlements^38^; therefore, we generated two distance-based rasters to characterize effects of human developments from TIGER/Line shapefiles representing (1) human settlements and (2) gravel and paved roads^50^. We processed all raster imagery using Google Earth Engine^46^.

### Preliminary covariate selection

We summarized explanatory data layers into multiple candidate covariates (Table 1). Ecological patterns can be sensitive to the scale of measurement^51^, so we considered six spatial scales when suitable to identify a spatial scale that was most influential for each covariate on plover breeding densities. The candidate spatial scales we considered ranged from site- to landscape-level which were: (1) at the 30 m pixel, (2) neighboring pixels (all 30 m pixels adjacent to the focal 30 m pixel), (3) 90 m radius, (4) 150 m radius, (5) 300 m radius, and (6) 750 m radius. We summarized varying spatial scales (radii) via a moving window analysis. For slope covariates, we summarized multiple pixels using their standard deviation and for all other covariates, we summarized multiple pixels using their average value. All candidate covariates were scaled to have a mean of 0 and standard deviation of 1. For effects of roads or human settlements, we considered the distance to these features in addition to a simpler binary classification of within 1 km of settlements or 100 m of roads.

While an objective of this research was to evaluate environmental associations with plover breeding habitat use and densities, an additional objective was to create annual spatially explicit predictions, which could be used to inform conservation and management of the species. Therefore, we used a statistical regularization framework to optimize model selection for predictive ability rather than strictly inference^14,52^. We used the least absolute shrinkage and selection operator (LASSO)^53^ as a method for variable selection to determine a final model for predictions. We fit LASSO models using a downweighted Poisson generalized linear regression to approximate an inhomogeneous Poisson point process with a set of 200,000 random points distributed across our study area to serve as quadrature locations^27^. Preliminary sensitivity analyses indicated that 200,000 points were sufficient for convergence of the log-likelihoods^27^. To select the optimal spatial scale for each covariate, we ran univariate linear models with the regularization penalty set to 0. Once covariates were scale-optimized using the smallest RMSE, we ran five global LASSO models to determine the most-supported form of related variables (e.g., binary versus continuous predictors and continuous versus exponential predictors; Table S1). We hypothesized that certain covariates would have nonlinear effects on plover breeding densities (e.g., intermediate levels of surface water may be preferred), therefore we tested a quadratic effect on percent water and NDVI. None of the predictor variables in each of the five global models were highly correlated (|r|> 0.70). We evaluated predictive performance of each candidate model by partitioning the data into 10 random subsets and using cross validation to calculate root mean squared errors (RMSE) and area under the receiver operating characteristic curve (AUC). A list of candidate models and their associated performance measures are in Table S1. Variable selection and cross validation with LASSO were conducted with the *cv.glmnet* function in the glmnet package^54^ in the R programming environment^55^, using methods described by Gerber and Northrup^14^. We conducted this variable selection process on nest locations alone because this process did not allow for the estimation of a joint-likelihood with eBird observations.

### Integrated spatiotemporal model

To predict the density of breeding plovers across the PPR, we used an integrated species distribution model^21^ which was fit using integrated nested Laplace approximation in R (R-INLA)^56^. INLA is an efficient alternative to Markov chain Monte Carlo for estimating Bayesian inference and avoiding convergence issues that are often associated with large spatiotemporal data^56^. Species distributions are commonly modeled as a log-Gaussian Cox process^57^, which can be computationally expensive. We modeled this process in INLA using stochastic partial differential equations (SPDE)^58^ to model spatial and temporal autocorrelations across a triangular mesh. We used SPDE to model a spatial random field with mean 0 and a Matérn covariance function^58^. To define the mesh design and triangle size, we started with a low-resolution mesh and iteratively fit smaller triangles to identify an optimal resolution that balanced computational cost and quality of approximation^59^. Modeling temporal variation in species distributions can be achieved by allowing environmental associations or residual spatial variation to fluctuate over time. Therefore, we modeled temporal autocorrelation with an autoregressive AR1 process for residual annual error.

Because we lacked information about true absences from nest site data, we modeled both datasets as presence-only locations. For nests, the intensity defined the expected number of points at location *s* and year *t*: $\lambda \left(s,t\right)= {e}^{\eta (s,t)}$. The latent plover distribution was modeled as a space–time inhomogeneous Poisson point process^60^. The intensity varied as a function of ecological covariates ***X***(*s*), and a random spatiotemporal field *u*(*s,t*) to account for unmeasured covariates and spatiotemporal autocorrelation:

$$\eta \left(s,t\right)= \sum_{i=1}^{N}\beta {{\varvec{X}}}_{i}\left(s\right)+u\left(s,t\right)$$

and *β* was a vector of regression coefficients. We assumed eBird observations emerged from a thinned intensity surface^21,61^. eBird observations were treated as a thinned-out version of the complete distribution of plover individuals in our study area, with survey effort and site accessibility covariates ***Z***(*s*) on the observation process (Table 1). Covariates for survey effort included checklist distance and duration, and these were obtained from eBird metadata. To account for site accessibility, we used TIGER/Line shapefiles representing roads^50^ and calculated the density of roads within 5 km of eBird points. The thinned latent species distribution was modeled with intensity $\lambda \left(s,t\right)b(s)$, where $b(s)$ was the thinning probability:

$$\mathrm{log}\left(\lambda \left(s,t\right)b\left(s\right)\right)= \sum_{i=1}^{N}\beta {{\varvec{X}}}_{i}\left(s\right)+u\left(s,t\right)+ \sum_{j=1}^{Q}\delta {{\varvec{Z}}}_{j}\left(s\right)$$

and *δ* was a vector of coefficients on the observation process. We used default settings in R-INLA for non-informative prior distributions for fixed effect coefficients, which were normally distributed with mean of 0 and precision of 0.001. We used a penalized complexity prior for the spatiotemporal random effects^62^ and informed a nominal range for the SPDE mesh based on the distance at which residual autocorrelation declined to approximately 0.1^63^.

### Spatial predictions

We generated spatial predictions at a 30 m resolution for each year of our study as an exponential function of ecological explanatory layers and the estimated year-specific random field. We summarized annual layers by delineating the study area as high or low habitat suitability to serve as a rapid assessment tool in addition to continuous predictions. We calculated sensitivity and specificity and identified a habitat suitability threshold value using the Symmetric Extremal Dependence Index (SEDI)^64^. SEDI is an evaluation metric that was developed for meteorological studies but has been shown to perform well in evaluating species distribution models when the frequency of presence points is low^65^. For our purposes, the habitat suitability threshold represented a practical yet conservative threshold at which plover presence was likely. We summarized annual layers based on the expected intensity of plover presence points and interpreted these values as relative indices of plover abundance^27^. We summed all cell values that were greater than the habitat suitability threshold in each year across our study area to represent relative plover abundance. We calculated bootstrapped 95% confidence intervals (CI) around plover abundance by recalculating abundance 10,000 times based on random samples of the data.

### Model validation

Because validating integrated models using data with different quality and observation processes is not always straightforward^21,66^, we used multiple approaches to assess prediction error. Evaluating the predictive performance of a species distribution model is best accomplished with out-of-sample data that are not used to estimate parameters^67^. Therefore, we additionally generated spatial prediction surfaces for 2020 and 2021 with explanatory layers from those years and an averaged (across the study period) spatial random field. We evaluated these predictions against eBird observations from 2020 and 2021 to serve as an out-of-sample model validation. The point accuracy of eBird observations was likely coarser than 30 m resolution predictions (i.e., exact locations of observed plovers were not recorded). Therefore, we buffered each eBird checklist using the survey distance to represent the area that was surveyed. We then used these buffers to validate 2020 and 2021 predicted surfaces using the maximum raster value that fell within the buffer (i.e., if any pixel within a buffer had high predicted intensity, the model would be considered accurate in that instance).

Cross-validation techniques can also be used to approximate the predictive ability of a model when out-of-sample data are sparse or unavailable^68^. Randomly selecting subsets of the data may underestimate model error because it does not force the model to extrapolate into new spatiotemporal structures^17,18,68^. Further, the nesting data we used to identify a final model for predictions were spatially restricted to a portion of the entire region of interest. Therefore, we considered both temporal and spatial block cross validation to assess these structures within our data^68^. For the temporal cross validation, we partitioned the eBird and nesting data into 3 time periods (2000–2006, 2007–2012, and 2013–2019; Fig. S1). For spatial block cross validation, we considered two scenarios: partitioning the data into 3 sets of 100 km blocks and 5 sets of 50 km blocks. We used the *blockCV* package in R to create spatial blocks^69^. Block sizes were selected based on the effective range of spatial autocorrelation in the explanatory data layers by fitting variograms to continuous rasters^69^. Lastly, we compared models with and without eBird observations using random tenfold cross validation to assess if predictive ability improved with the inclusion of eBird data in addition to the nest locations. We compared out-of-sample and cross validation strategies using RMSE and AUC.

## Results

Nest locations were collected in North Dakota and eastern Montana, with no spatial coverage in South Dakota (*n* = 4,621). The average number of nest locations collected per year was 231 (range = 120–386; Fig. S1). We included 487 eBird records in our analysis that were collected between 2000 and 2019 and these points had spatial coverage throughout the study region (Fig. 1), thus filling spatial gaps from nest locations. The number of eBird records per year increased over time, particularly after 2013 (Fig. S1).

We conducted a preliminary covariate selection procedure using LASSO regularization and found that the optimal spatial scale of effect varied among covariates (Table S1). Slope and NDVI were most supported at the 30 m pixel scale, whereas the percentage of surface water (DSWE) was most supported with a 90 m moving window. The effect of crop and hay pasture was most supported at the scale of neighboring pixels. We found that LASSO regularization consistently removed the effect of distance to trees by constraining it to zero, therefore we generated an additional model without this parameter. The final model with the most support and best predictive ability included percent water (90 m scale), NDVI (30 m scale), slope (30 m scale), crop and hay pasture (neighbor scale), distance to lakes as an exponential function, 100 m to roads (binary), and 1 km to settlements (binary) (Table S1). Further, we found support for quadratic effects for percent water and NDVI.

The final integrated point process model included seven ecological covariates and three observation covariates (Table 2). Parameter estimates for site accessibility and checklist effort indicated that eBird points increased in intensity with greater road density, and checklist duration and distance, although 95% credible intervals overlapped 0 for both checklist effort covariates. Plover breeding intensity was highest when the percentage of surface water and NDVI values were both low, but not at their minimum values (Table 2). We found a strong negative relationship between the distance to lakes and breeding intensity, where intensity was highest close to lakes. Roads and settlements had similar effects, where plover breeding intensity was lower near these features (Table 2).

**Table 2:** Log-scale posterior median effect sizes and Bayesian credible intervals of a multiscale integrated point process model for breeding piping plovers.

Parameter	2.5%	50.0%	97.5%
Ecological model			
Intercept	− 1.91	− 1.90	− 1.88
Slope	− 0.42	− 0.40	− 0.39
NDVI	0.07	0.08	0.09
NDVI^2^	− 0.23	− 0.22	− 0.21
Crop and hay pasture	− 0.54	− 0.52	− 0.49
Percent surface water	2.22	2.24	2.27
Percent surface water^2^	− 0.40	− 0.39	− 0.38
Dist. to lake (exponential)	− 9.44	− 9.40	− 9.37
Settlement (> 1 km from settlements = 1, < 1 km from settlements = 0)	0.15	0.18	0.20
Road (> 100 m from roads = 1, < 100 m from roads = 0)	0.61	0.64	0.67
eBird observation model			
Effort distance	− 0.08	0.02	0.10
Effort duration	− 0.06	0.04	0.05
Road density	0.11	0.12	0.22

Slope and normalized difference vegetation index (NDVI) were measured at the 30 m pixel scale, crop and hay pasture were measured with a mean of neighboring pixels moving window analysis, and percent surface water was measured with a mean of 90 m moving window analysis.

**Figure 2:**
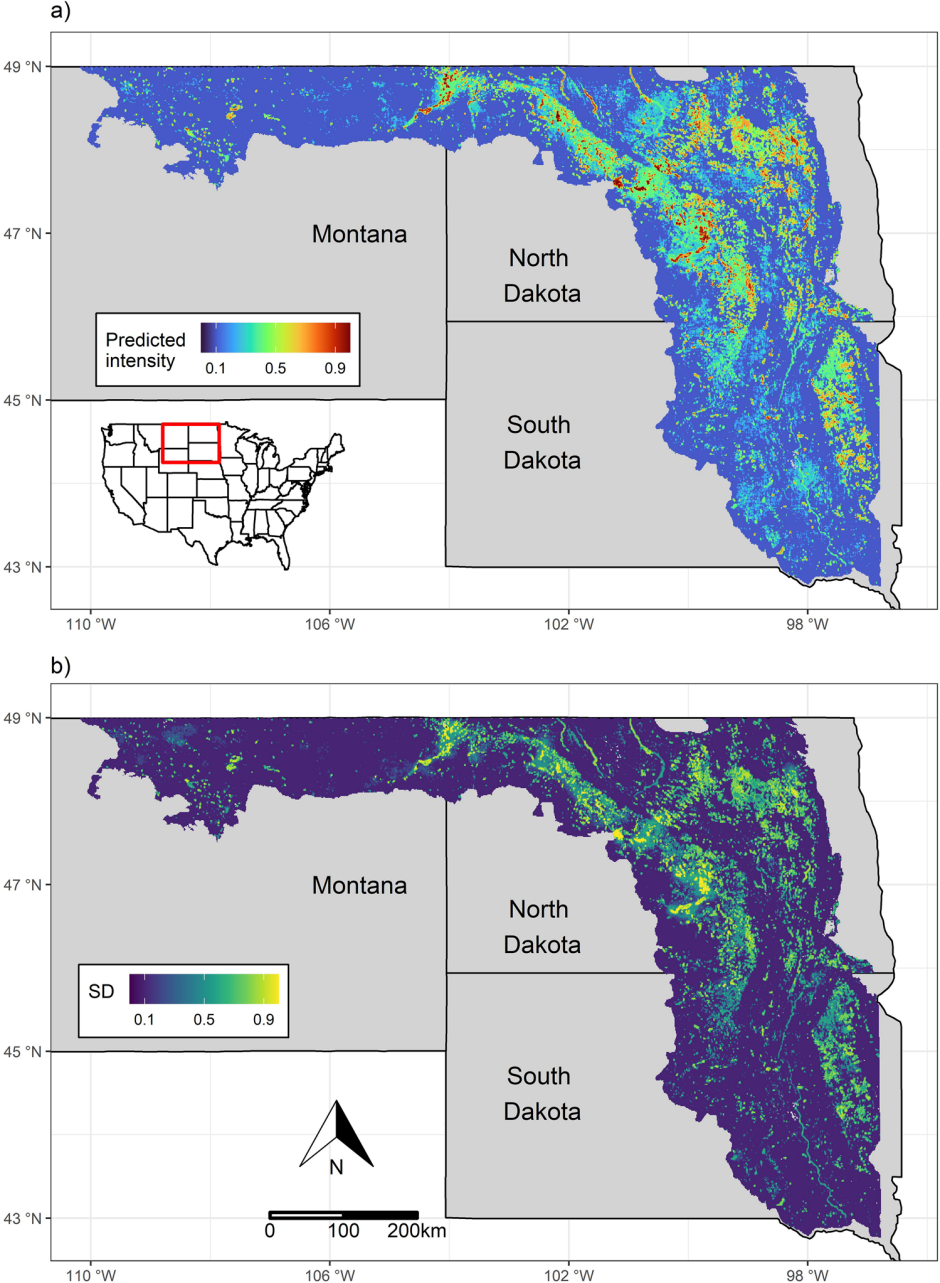
Average (**a**) and standard deviation (**b**) of predicted piping plover (*Charadrius melodus*) breeding habitat use and density across our nest data collection period (2000–2019). To allow for improved visualization, maps were aggregated to 900 m resolution using the mean of pixel values and normalized by rescaling values between 0 and 1. Maps were generated using R (version 4.1.3)^55^.

**Figure 3:**
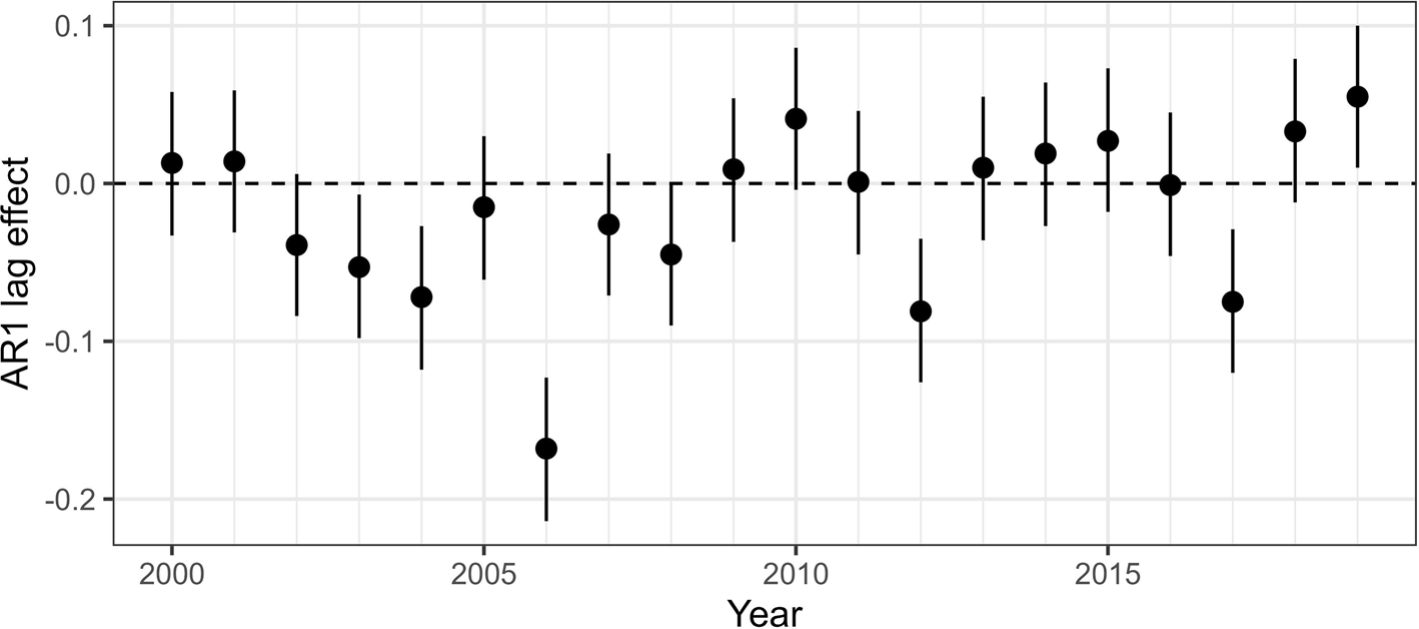
Log-scale median estimates with 95% credible interval of annual autocorrelation in point intensity (autoregressive, AR1 lag effect). The dashed line indicates a lag effect of 0.

**Figure 4:**
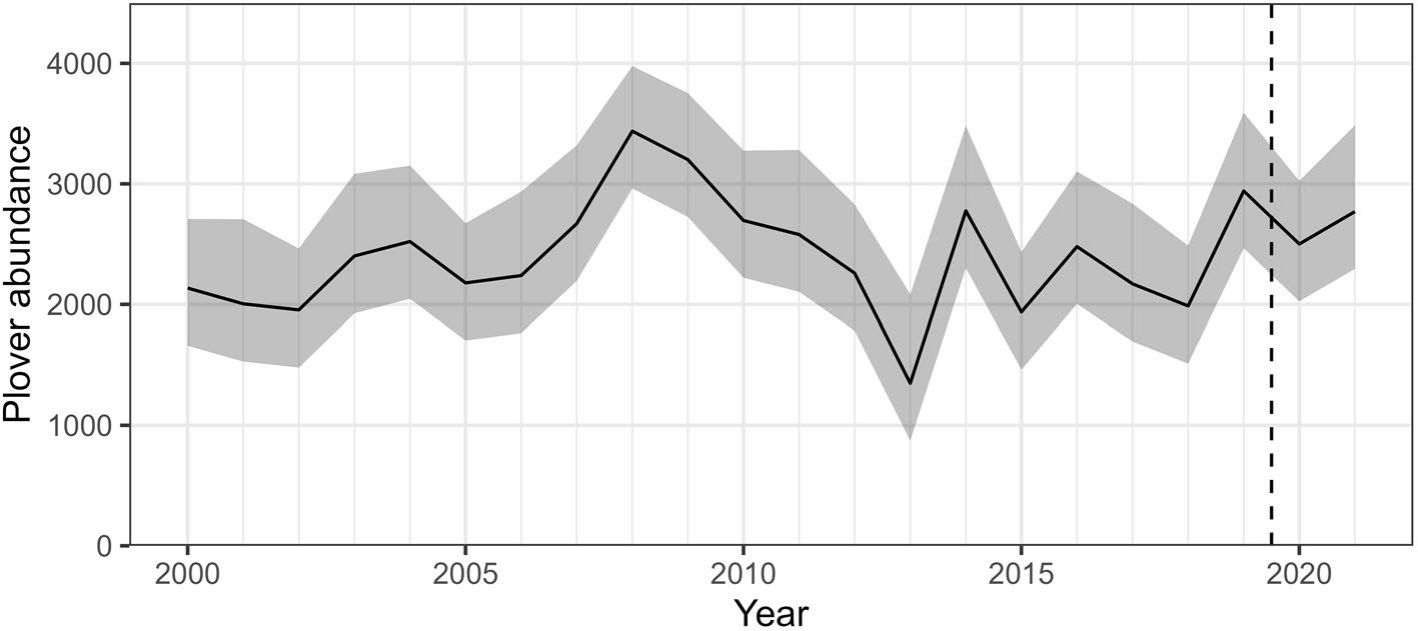
Piping plover (*Charadrius melodus*) relative abundance (sum of predicted point intensities) across the U.S. Prairie Pothole Region in each year. Estimates were calculated using pixels that were greater than the habitat suitability threshold. Estimates from 2020 and 2021 were based on an average spatial random field and habitat associations generated from nesting data collected between 2000 and 2019 (denoted by the dashed vertical line). The shaded area indicates 95% confidence intervals.

We used an SPDE mesh with 63,840 vertices. The random spatiotemporal field predicted hotspots of high intensity (visually related to nest or eBird clusters) that covariates alone did not explain (Fig. 2, Fig. S3). The median nominal range of spatial autocorrelation was 116 km (95% CI: 104–130 km). Annual autocorrelation of point intensities was high (0.94, 95% CI: 0.92–0.95) yet there was variation across years in the spatial distribution and intensity of predicted nesting habitats (Fig. 3, Figs. S4, S5). The predicted plover abundance (point intensity) across the PPR averaged 2,417.48 (95% CI: 1,657.67–3,313.27, total prediction area = 235,464.50 km^2^; Fig. 4).

The mean AUC across spatial and temporal block cross-validation procedures was greater than 0.80 (Table S2), and RMSE metrics were in general agreement with AUC. Model accuracy was similar between 3- and 5-block spatial cross-validation (3-block AUC = 0.83, 5-block AUC = 0.82, 3-block RMSE = 0.22, 5-block RMSE = 0.28; Table S2, Fig. S6), whereas the highest model accuracy was associated with temporal cross validation (AUC = 0.97, RMSE = 0.14). Out-of-sample model validation using exclusively eBird locations from 2020 and 2021 (*n* = 187) had the lowest model accuracy (AUC = 0.64, RMSE = 0.36), compared to cross-validation approaches that used a combination of nest and eBird locations (Table S2). Random tenfold cross validation indicated that excluding eBird locations from the model reduced prediction accuracy (RMSE = 0.15 with eBird and 0.42 without eBird; Table S2).

## Discussion

Spatial and temporal patterns of breeding distributions in dynamic landscapes can be highly variable for many migratory bird species^31,70,71^. We applied an integrated species distribution model and predicted breeding habitat use and density of an uncommon species across broad spatial and temporal extents. Our model had greater temporal transferability than spatial transferability, indicating that predictions are likely to be more robust when extrapolating to new time periods compared to new spatial regions. Collecting annual nesting data is costly, therefore natural resource managers may benefit from using spatial predictions as an inexpensive tool for estimating annual breeding habitats and relative abundances, or for directing sample-based monitoring efforts. While spatial predictions can be informative, there are still uncertainties regarding the reliability of predictions when forecasting based on our 20-year nesting dataset, as climate and future land use changes will likely continue to alter plover habitats in the PPR^47^. Such uncertainties indicate that continuing to collect nesting data, while expanding efforts outside of the core nesting regions will likely be valuable in assessing temporal limitations of our predictions. Further, our results did not indicate that eBird observations served as a robust alternative to nesting locations during our study period, although model fit was better than a random sample. Therefore, relying on these data exclusively in the future would benefit from further assessment.

Preliminary filtering of eBird data can be useful for removing outliers but is often inadequate at completely accounting for observational noise^25,26^. Of the observational covariates, road density had the greatest effect, indicating that site accessibility was more influential than survey effort on eBird plover sightings. Many regions within our study extent were on private lands and distant from large population centers, and community science data can often be clustered around popular locations with established access and human settlements^25,26^. Despite these observational biases, the benefit of including the eBird database in our study was that these sightings had greater spatial coverage than nest monitoring efforts, thus providing an improved basis for prediction outside the spatial footprint of the monitoring data.

Assessing model fit when using multiple streams of data in an integrated framework is often not straightforward^21,59,66^. Our process for measuring model error was to use both out-of-sample data and cross-validation approaches. While we did not generate a model using exclusively eBird data, we suspect that it would have been difficult to extract reliable ecological signals because out-of-sample model validation using only eBird observations showed the lowest agreement with model predictions. An explanation for the reduced accuracy we observed may be that many eBird points fell outside of core nest monitoring regions (i.e., hotspots generated by the 20-year spatial random effect). eBird observations outside of nesting hotspots would have been in areas with a lower predicted breeding intensity, which is presumably indicative of true patterns in this system (plover breeding intensity is not evenly distributed throughout the PPR). While community science data are typically plentiful, the number of eBird observations across the PPR was low in the earlier years of our study and their contributions to the spatial random effect in those years were likely minimal. This out-of-sample validation additionally indicates that there may be a loss in model accuracy associated with relying on the 20-year averaged spatial effect compared to year-specific effects. There are numerous complexities when determining what types of data to include, yet a general philosophy of integrated modeling is to take advantage of the maximum amount of information^21,22^.

Plover breeding densities in the PPR showed high spatial and temporal autocorrelation. Accounting for this autocorrelation carries distinct advantages over non-explicit alternatives when modeling mobile species that select habitats based on behavioral or other cues of the environment that can be difficult to account for^31,59^. Avian species may shift their breeding distributions across time to take advantage of suitable habitats^71,72^, yet high site fidelity can lead to greater reproductive success^73^ or population persistence^74^ when site quality is temporally correlated. However, rapid environmental changes may also lead to instances where high site fidelity is disadvantageous because species’ abilities to respond to habitat alteration can be relatively slow^75^. Plovers typically show high site fidelity across their range, and rates can vary annually and as a function of age, sex, or prior reproductive success^37,76–79^. There are tradeoffs when considering the computational costs of processing annual datasets and generating dynamic predictions versus the loss in precision that may be associated with a long-term representation of breeding distributions. Understanding variation in site fidelity or factors influencing dispersal probabilities and distances could provide a basis for when dynamic predictions may be preferred over static predictions for management and conservation decisions, particularly when habitat availability varies. For example, distances between individual plover nesting sites across successive breeding seasons were greater when other known breeding areas were farther away^80^. This pattern indicates that when breeding areas are more isolated or disjointed, spatiotemporal autocorrelation in plover density may be lower and breeding densities may therefore be more dependent on the availability of habitats. In these instances, dynamic habitat predictions may be more informative than a long-term average.

Our model of plover breeding habitat use and density was informed by dynamic data layers, including surface water, vegetation coverage, and land use, yet distance to permanent lakes was also supported by the data as a static measurement that was invariant across time. Shorebird distributions have previously been shown to depend on both dynamic and long-term environmental conditions^3^, consistent with relationships observed widely across avian taxa^70^. The PPR typically experiences cyclic precipitation patterns with drought and wet phases, which can influence water levels in prairie wetlands^81^, and subsequently the abundance and distributions of birds using these habitats^10,32,82^. Moreover, water-level dynamics in the PPR contribute to the maintenance of unvegetated shorelines^83^, which are essential for breeding activities of plovers and other shorebird species in the region^33^. Land use practices in the PPR have promoted fewer, but larger and fuller wetlands that do not draw down as easily^84^, and fuller wetlands are associated with a lower probability of plover presence^47^. Future climate change projections in the PPR predict wetter conditions during the spring and summer, and warmer temperatures may promote longer growing seasons and vegetation encroachment^85^. Therefore, plovers breeding in prairie wetlands may be highly sensitive to climate change because of their requirements for unvegetated, shallow wetlands. While we found that the predicted abundance and density of nests varied with habitat availability, we did not detect a consistent increasing or decreasing trend throughout our study, despite an increase in the quantity of plover eBird observations.

Plovers and other shorebirds in the PPR use widely dispersed wetlands that often occur on private lands where agriculture is a dominant land use^10,33^. Conservation and land use planning to accommodate dynamic habitat changes in the PPR would require planning in a highly uncertain future over a broad spatial extent^10,47^. Therefore, dynamic and long-term predictions of plover breeding intensity may have separate value for conservation and management decisions, in terms of when and where suitable habitats occur. However, portions of the PPR remain understudied and additional sampling in areas where habitats are intermittently available would aid in assessing the magnitude of change in breeding density outside of the core monitoring areas, and potentially the ability of plovers to adapt to climate changes. Annual predictions generated from our model could serve as a tool for directing monitoring efforts to unmonitored regions with suitable habitats. Future research that focuses on past and future climate conditions to identify regions that are likely to be robust for concentrating conservation actions will likely provide a basis for developing effective long-term management strategies^85^.

## Supplementary Information

Supplementary Information.

## Data Availability

Nesting data generated during this study are available as a USGS data release (https://doi.org/10.5066/P9FZJZXU). Remotely sensed data layers can be acquired using Google Earth Engine code included in the Supplemental Materials. eBird data are publicly available (eBird.org).
