# Supplementary material for: Data integration reveals dynamic and systematic patterns of breeding habitat use by a threatened shorebird
Source: Sci Rep. 2023 Apr 13;13:6087. doi: 10.1038/s41598-023-32886-w (PMC10102276; doi:10.1038/s41598-023-32886-w)
Supplement: Supplementary file 1 — Supplementary Information. [file 41598_2023_32886_MOESM1_ESM.pdf]

Supplementary materials for: Data integration reveals dynamic and systematic patterns of breeding habitat use by a threatened shorebird

Kristen S. Ellis[1]*, Michael J. Anteau[1], Garrett J. MacDonald[1], Rose J. Swift[1], Megan M. Ring[1], Dustin L. Toy[1], Mark H. Sherfy[1], and Max Post van der Burg[1]

[1]U.S. Geological Survey, Northern Prairie Wildlife Research Center, 8711 37th St SE, Jamestown, ND 58401 USA

*Corresponding author email: kellis@usgs.gov

Table S1. Preliminary model selection for selecting an appropriate spatial scale of measurement and final combination of covariates using LASSO regularization. Bold spatial scales indicate the covariates with the highest support that were advanced to final modeling stages. The final model structure in bold was advanced to the integrated model. We evaluated predictive performance of each candidate model by partitioning the data into 10 random subsets and using cross validation to calculate root mean squared errors (RMSE) and area under the receiver operating characteristic curve (AUC). NDVI = Normalized Difference Vegetation Index.

**Model**	**RMSE**	**AUC**
Univariate scale selection		
Slope (moving window function: standard deviation)		
**30 m**	0.18 (0.05 – 0.24)	0.62 (0.55 – 0.71)
Neighbors	0.21 (0.11 – 0.36)	0.58 (0.51 – 0.61)
90 m	0.21 (0.08 – 0.36)	0.58 (0.51 – 0.61)
150 m	0.21 (0.16 – 0.31)	0.58 (0.51 – 0.61)
300 m	0.21 (0.08 – 0.36)	0.58 (0.51 – 0.61)
750 m	0.22 (0.08 – 0.37)	0.59 (0.50 – 0.61)
Percent surface water (moving window function: mean)		
30 m	0.15 (0.09 – 0.22)	0.78 (0.71 – 0.79)
Neighbors	0.15 (0.09 – 0.19)	0.79 (0.70 – 0.82)
**90 m**	0.13 (0.10 – 0.16)	0.86 (0.81 – 0.89)
150 m	0.14 (0.09 – 0.18)	0.85 (0.80 – 0.86)
300 m	0.14 (0.09 – 0.18)	0.81 (0.78 – 0.82)
750 m	0.14 (0.08 – 0.19)	0.80 (0.77 – 0.82)
NDVI (moving window function: mean)		
**30 m**	0.08 (0.03 – 0.11)	0.92 (0.88 – 0.95)
Neighbors	0.10 (0.05 – 0.18)	0.89 (0.81 – 0.90)
90 m	0.09 (0.04 – 0.21)	0.88 (0.80 – 0.91)
150 m	0.10 (0.05 – 0.17)	0.87 (0.80 – 0.89)
300 m	0.10 (0.05 – 0.17)	0.91 (0.81 – 0.94)
750 m	0.10 (0.05 – 0.18)	0.90 (0.82 – 0.94)
Crop and hay pasture (moving window function: mean)		
30 m	0.09 (0.01 – 0.17)	0.74 (0.69 – 0.78)
**Neighbors**	0.08 (0.01 – 0.12)	0.83 (0.75 – 0.89)
90 m	0.09 (0.03 – 0.22)	0.80 (0.74 – 0.86)
150 m	0.09 (0.03 – 0.19)	0.80 (0.74 – 0.86)
300 m	0.08 (0.02 – 0.19)	0.82 (0.77 – 0.85)

750 m	0.09 (0.03 – 0.21)	0.81 (0.72 – 0.88)
Final model selection		
slope + perc_water + perc_water^2 + NDVI + NDVI^2 + crop + dist_to_lake_exp + dist_to_roads + dist_to_settlement + dist_to_trees	0.93 (0.39 – 1.41)	0.95 (0.92 – 0.98)
slope + perc_water + perc_water^2 + NDVI + NDVI^2 + crop + dist_to_lake + dist_to_roads + dist_to_settlement + dist_to_trees	0.71 (0.18 – 1.65)	0.94 (0.85 – 0.95)
slope + perc_water + perc_water^2 + NDVI + NDVI^2 + crop + dist_to_lake_exp + road100m + settlement1km + dist_to_trees	0.65 (0.02 – 0.98)	0.97 (0.92 – 0.98)
slope + perc_water + perc_water^2 + NDVI + NDVI^2 + crop + dist_to_lake + road100m + settlement1km + dist_to_trees	0.69 (0.05 – 1.49)	0.95 (0.91 – 0.98)
**slope + perc_water + perc_water^2 + NDVI + NDVI^2 + crop + dist_to_lake_exp + road100m + settlement1km**	0.59 (0.06 – 1.16)	0.98 (0.97 – 0.99)

Table S2. Root mean square error (RMSE) and area under the receiver operating characteristic curve (AUC) with 95% confidence intervals for different model validation approaches. The number of nest and eBird point locations for piping plover, *Charadrius melodus*, that were partitioned into each testing set are also shown.

**Validation approach**	**Description**	**RMSE**	**AUC**	**Nest**	**eBird**
Out of sample	eBird points from 2020 and 2021	0.36 (0.16 - 0.84)	0.64 (0.63 - 0.69)	*n* = 0,<br>*n* = 0	*n* = 80,<br>*n* = 107
Temporal	3-block cross validation (2000 - 2006; 2007 - 2012; 2013 - 2019)	0.10 (0.09 - 0.15)	0.97 (0.96 - 0.98)	*n* = 1526,<br>*n* = 1780,<br>*n* = 1315	*n* = 98,<br>*n* = 92,<br>*n* = 297
Spatial	3-block cross validation (100 km blocks)	0.22 (0.04 - 0.41)	0.83 (0.78 - 0.86)	*n* = 1433,<br>*n* = 2291,<br>*n* = 897	*n* = 122,<br>*n* = 261,<br>*n* = 104
Spatial	5-block cross validation (50 km blocks)	0.28 (0.04 - 0.82)	0.82 (0.75 - 0.88)	*n* = 997,<br>*n* = 691,<br>*n* = 1311,<br>*n* = 321,<br>*n* = 1301	*n* = 64,<br>*n* = 116,<br>*n* = 90,<br>*n* = 97,<br>*n* = 120
Random	10-fold cross validation	0.15 (0.09 – 0.48)	0.98 (0.92 – 0.99)	*n* = 459,<br>*n* = 442,<br>*n* = 463,<br>*n* = 523,<br>*n* = 461,<br>*n* = 503,<br>*n* = 451,<br>*n* = 456,<br>*n* = 428,<br>*n* = 435	*n* = 46,<br>*n* = 54,<br>*n* = 56,<br>*n* = 51,<br>*n* = 49,<br>*n* = 44,<br>*n* = 50,<br>*n* = 50,<br>*n* = 47,<br>*n* = 40
Random – excluding eBird	10-fold cross validation	0.42 (0.23 – 0.77)	0.96 (0.91 – 0.98)	*n* = 459,<br>*n* = 442,<br>*n* = 463,<br>*n* = 523,<br>*n* = 461,<br>*n* = 503,<br>*n* = 451,<br>*n* = 456,<br>*n* = 428,<br>*n* = 435	*n* = 0,<br>*n* = 0,<br>*n* = 0,<br>*n* = 0,<br>*n* = 0,<br>*n* = 0,<br>*n* = 0,<br>*n* = 0,<br>*n* = 0,<br>*n* = 0

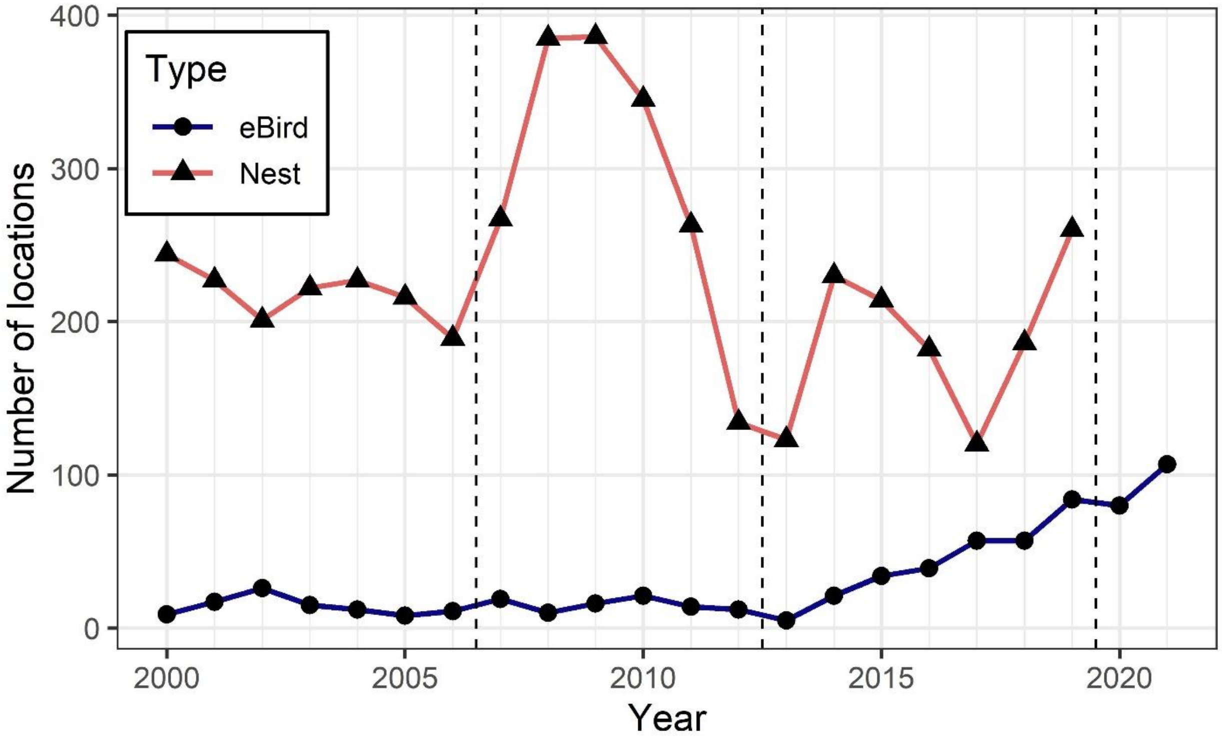

Fig. S1. The number of nest and eBird locations for piping plover, *Charadrius melodus*, in each year of our study. Dashed vertical lines indicate breaks in temporal subsets that were used for block cross validation (2000 – 2019). eBird locations from 2020 and 2021 were incorporated into model validation procedures as out-of-sample data.

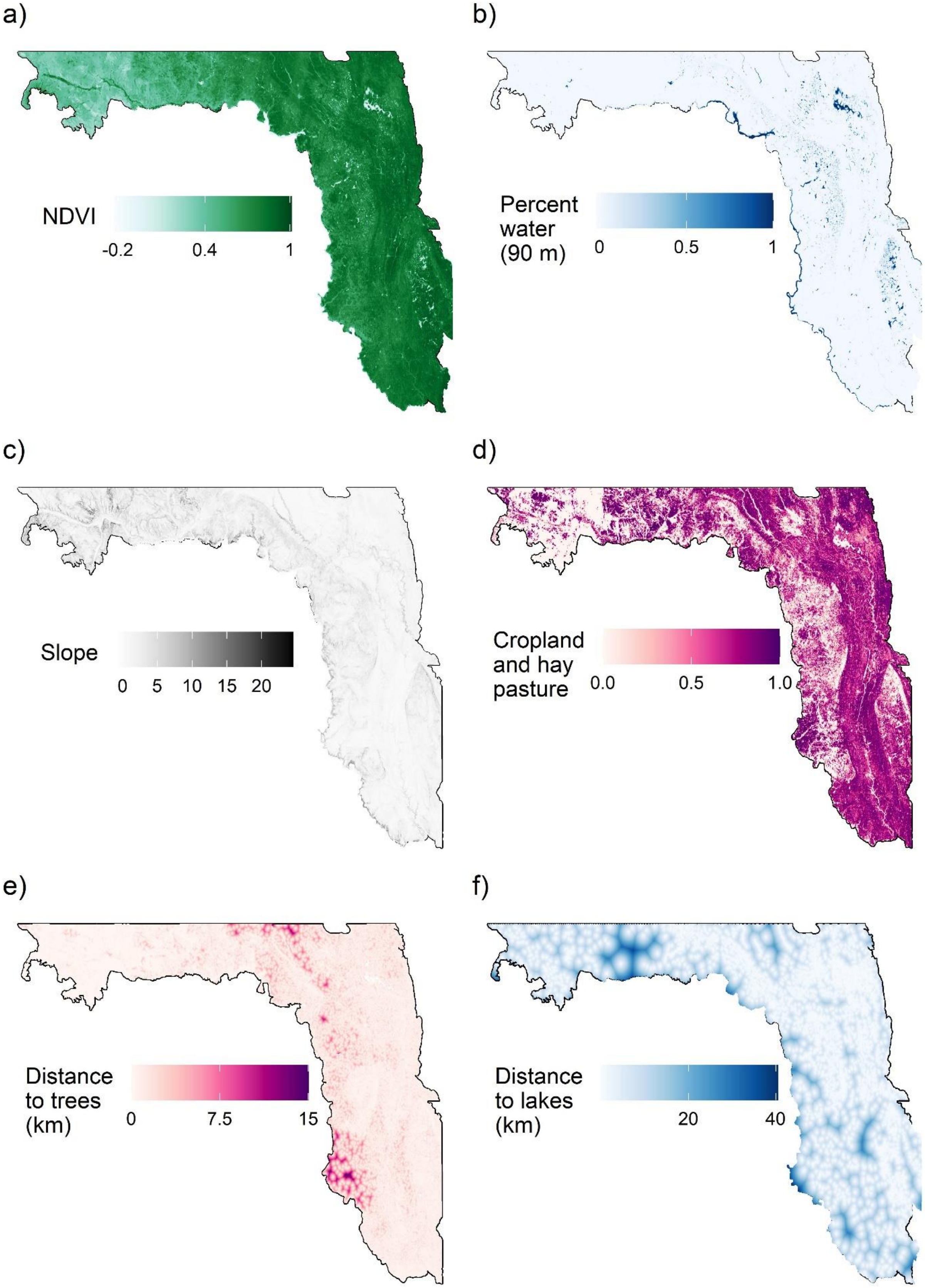
a)
NDVI
-0.2
0.4
1
b)
Percent water (90 m)
0
0.5
1
c)
Slope
0
5
10
15
20
d)
Cropland and hay pasture
0.0
0.5
1.0
e)
Distance to trees (km)
0
7.5
15
f)
Distance to lakes (km)
20
40

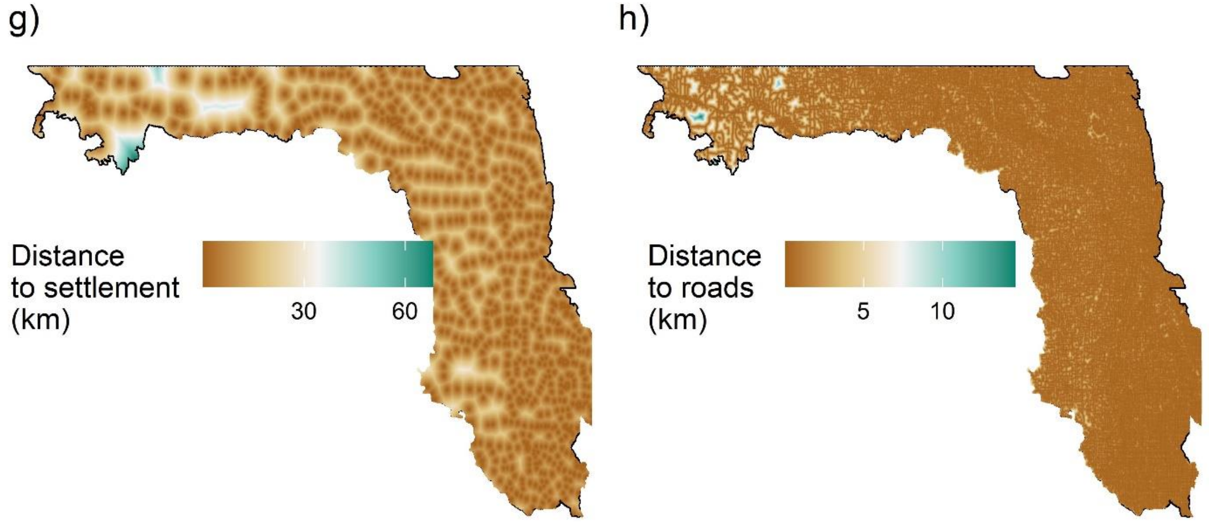

Fig. S2. Explanatory data layers that were used to generate ecological covariates for piping plover, *Charadrius melodus*. These layers included the Normalized Difference Vegetation Index (NDVI; a); percentage of surface water using the Dynamic Surface Water Extent (DSWE; b); degree of slope (c), cropland and hay pasture classified using the National Land Cover Database (NLCD; d); distance to trees from the NLCD (e); distance to National Wetland Inventory (NWI) lakes (f); distance to human settlements (g); and distance to gravel and paved roads (h). Plots a, b, d, and e represent averages across our study period (2000 – 2019). We did not consider the reservoirs of the Missouri River on the western boundary of our study area to calculate the distance to lakes shown in plot f. Maps were generated using R (version 4.1.3; www.r-project.org).

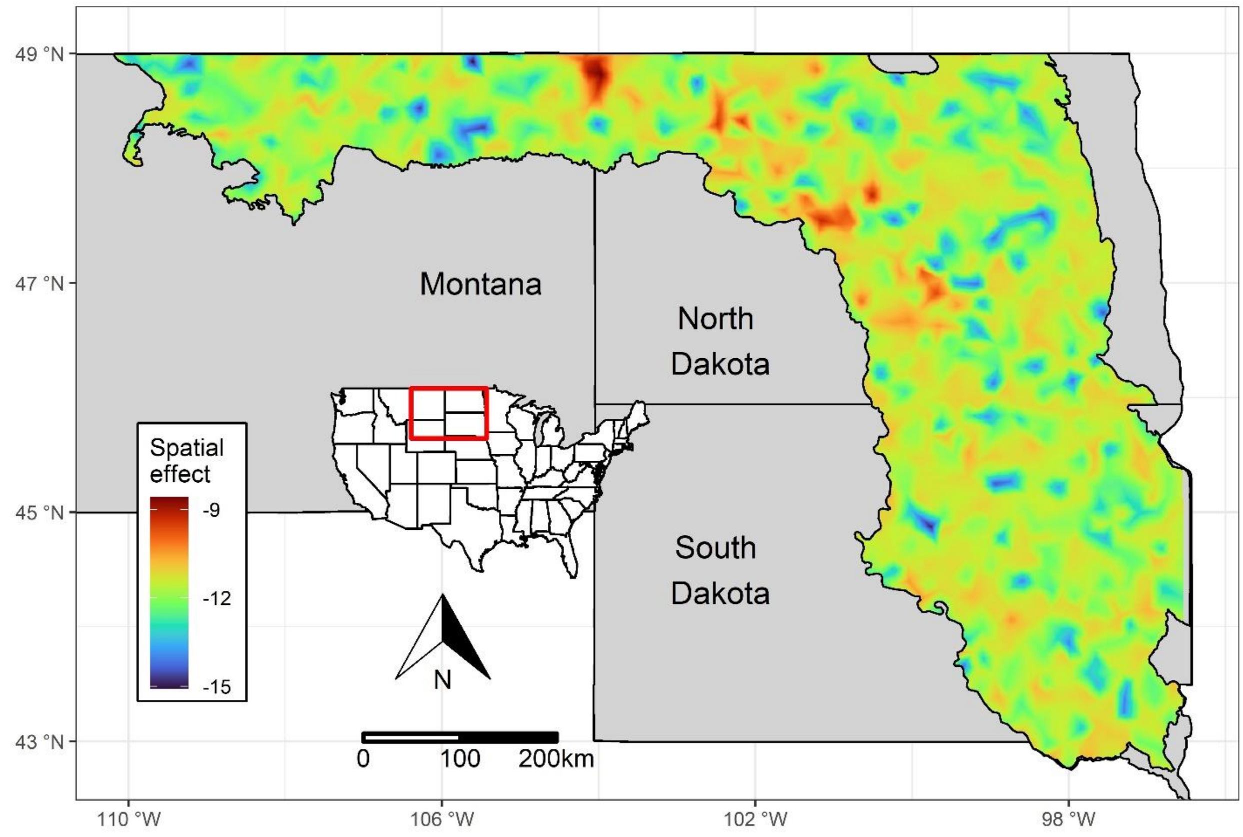

Fig. S3. Averaged spatial random effect across our nest collection period (2000 – 2019) for piping plover, *Charadrius melodus*. Temporal autocorrelation was modeled with an autoregressive AR1 error structure. The spatiotemporal random effect was generated using a mesh of 63,840 vertices. Maps were generated using R (version 4.1.3; www.r-project.org).

2000

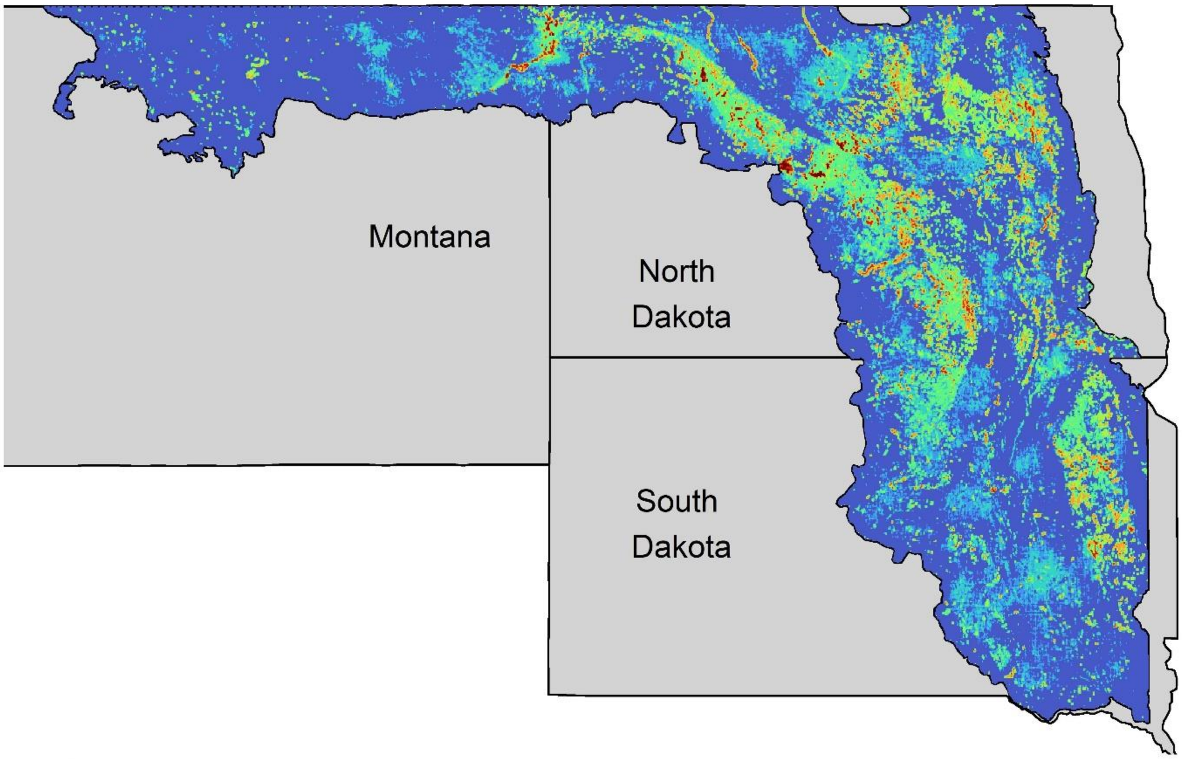
Montana
North Dakota
South Dakota

2001

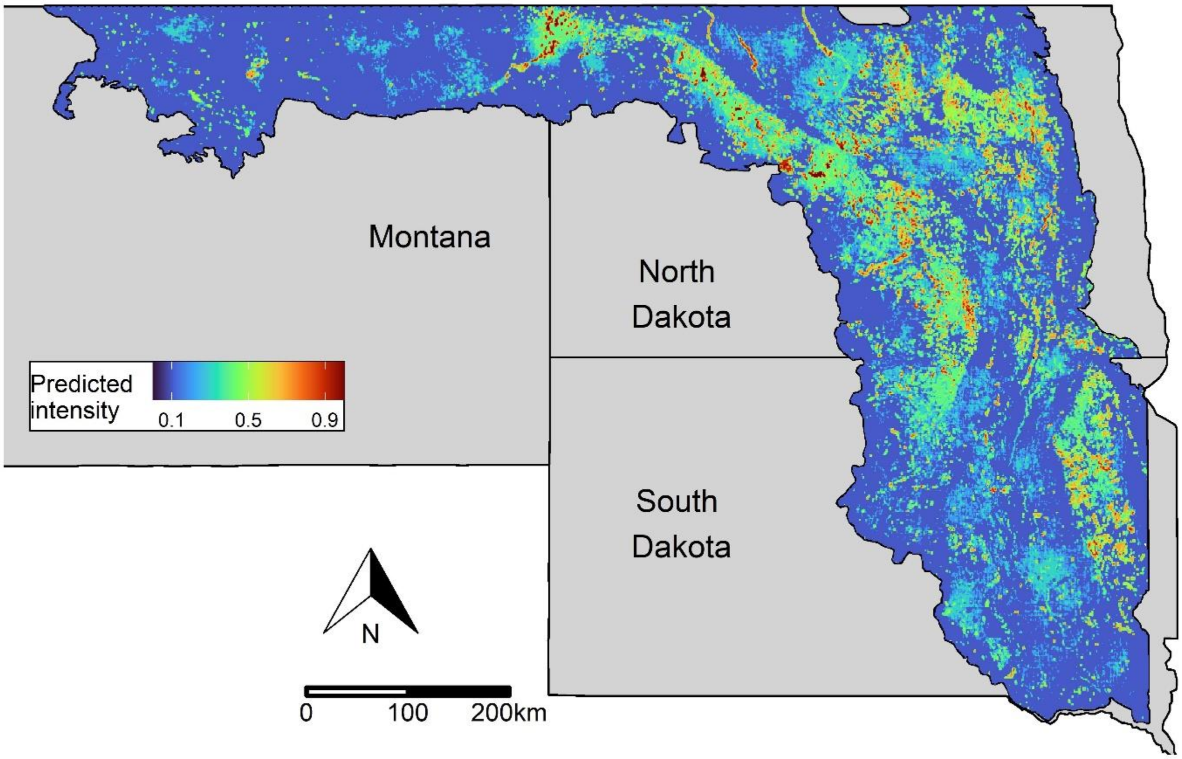
Montana
North Dakota
South Dakota
Predicted intensity
0.1
0.5
0.9
N
0
100
200km

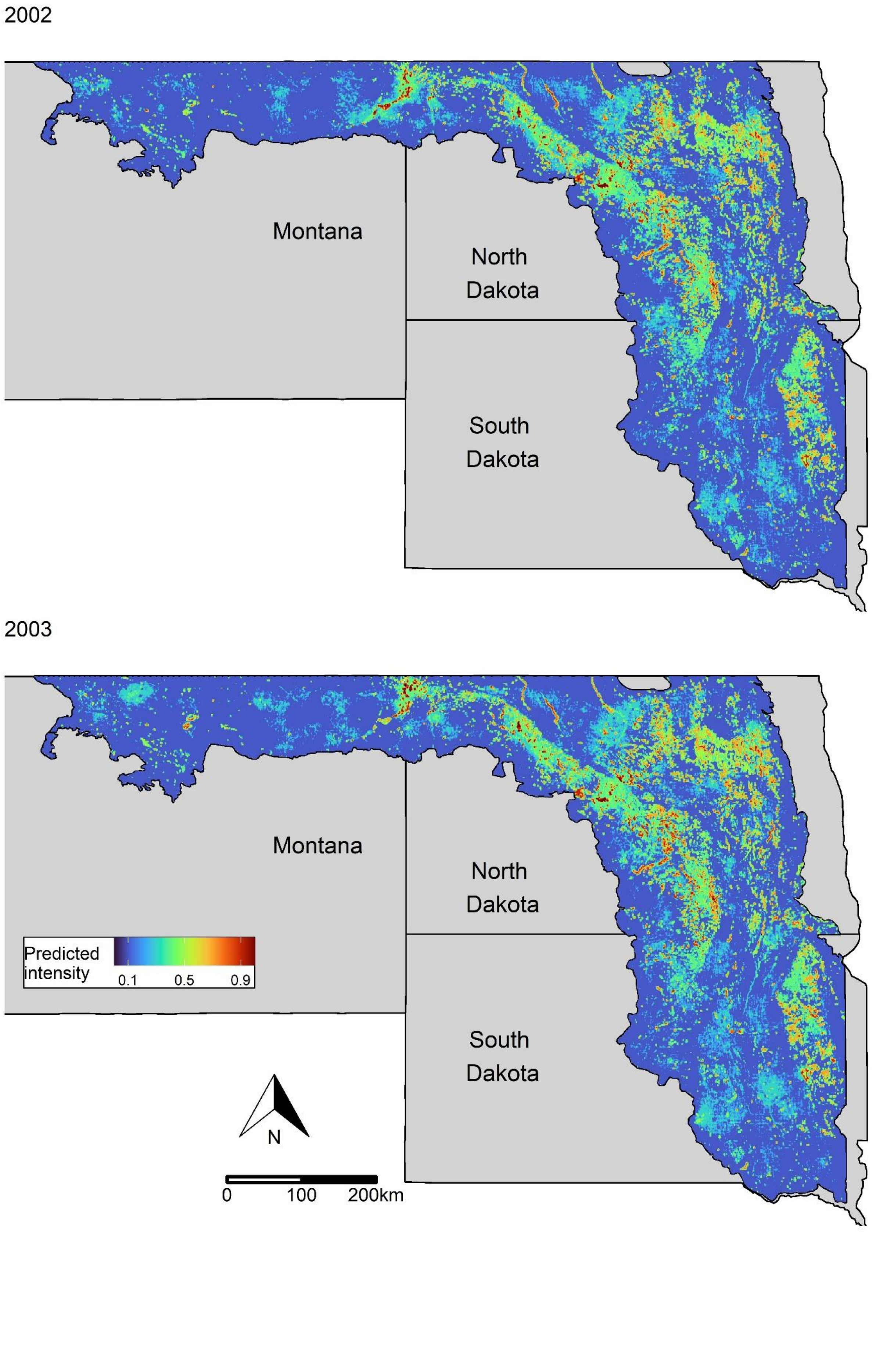
2002
Montana
North
Dakota
South
Dakota
2003
Montana
North
Dakota
South
Dakota
Predicted
intensity
0.1
0.5
0.9
N
0
100
200km

2004

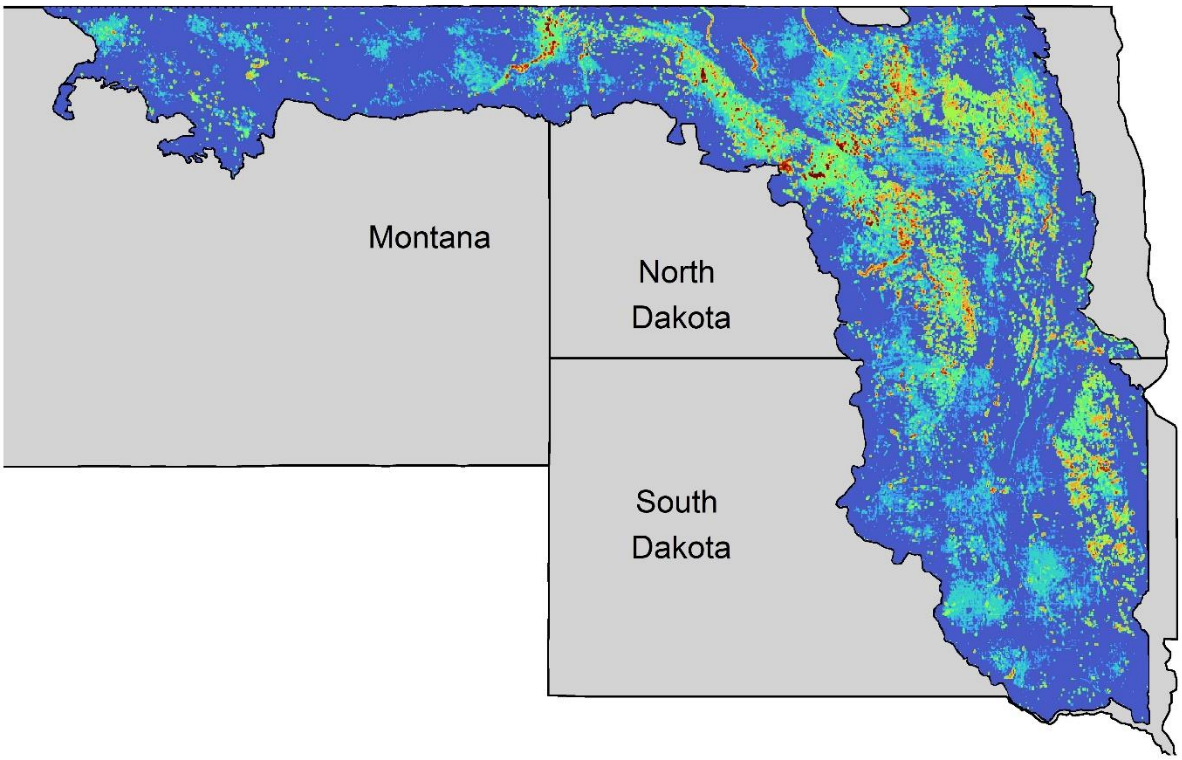
Montana
North
Dakota
South
Dakota

2005

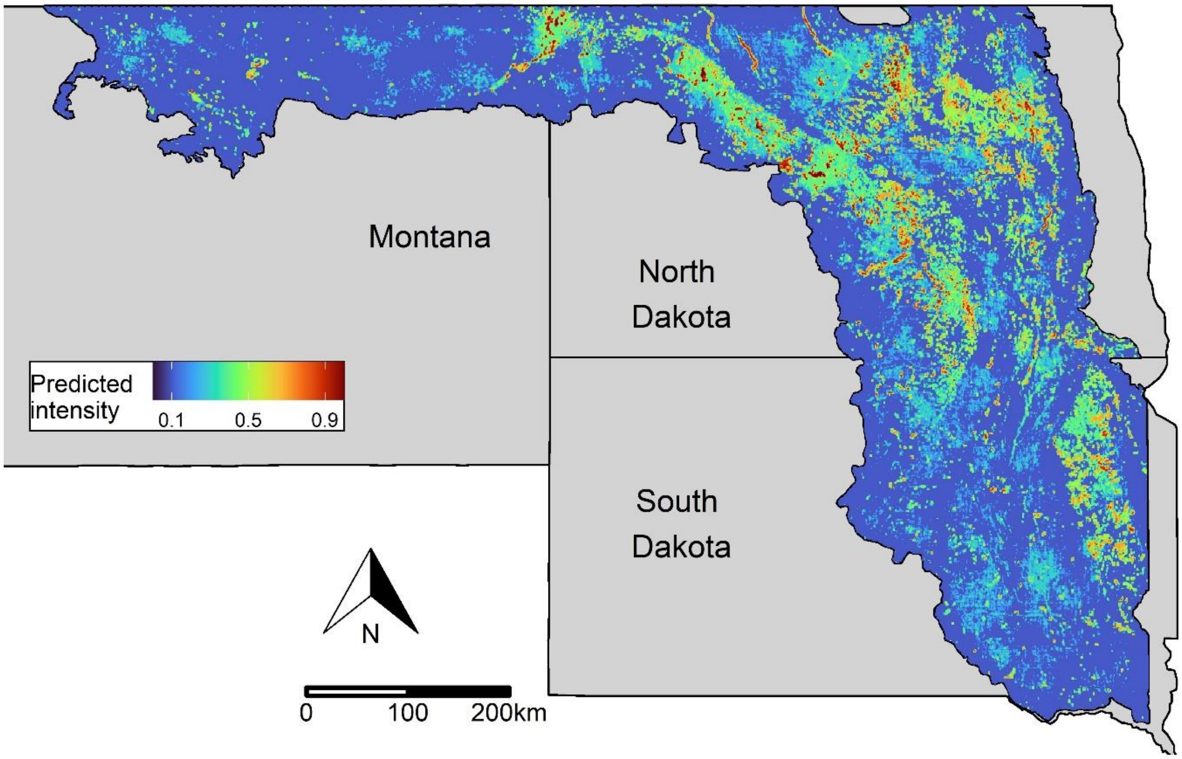
Montana
North
Dakota
South
Dakota
Predicted
intensity
0.1
0.5
0.9
N
0
100
200km

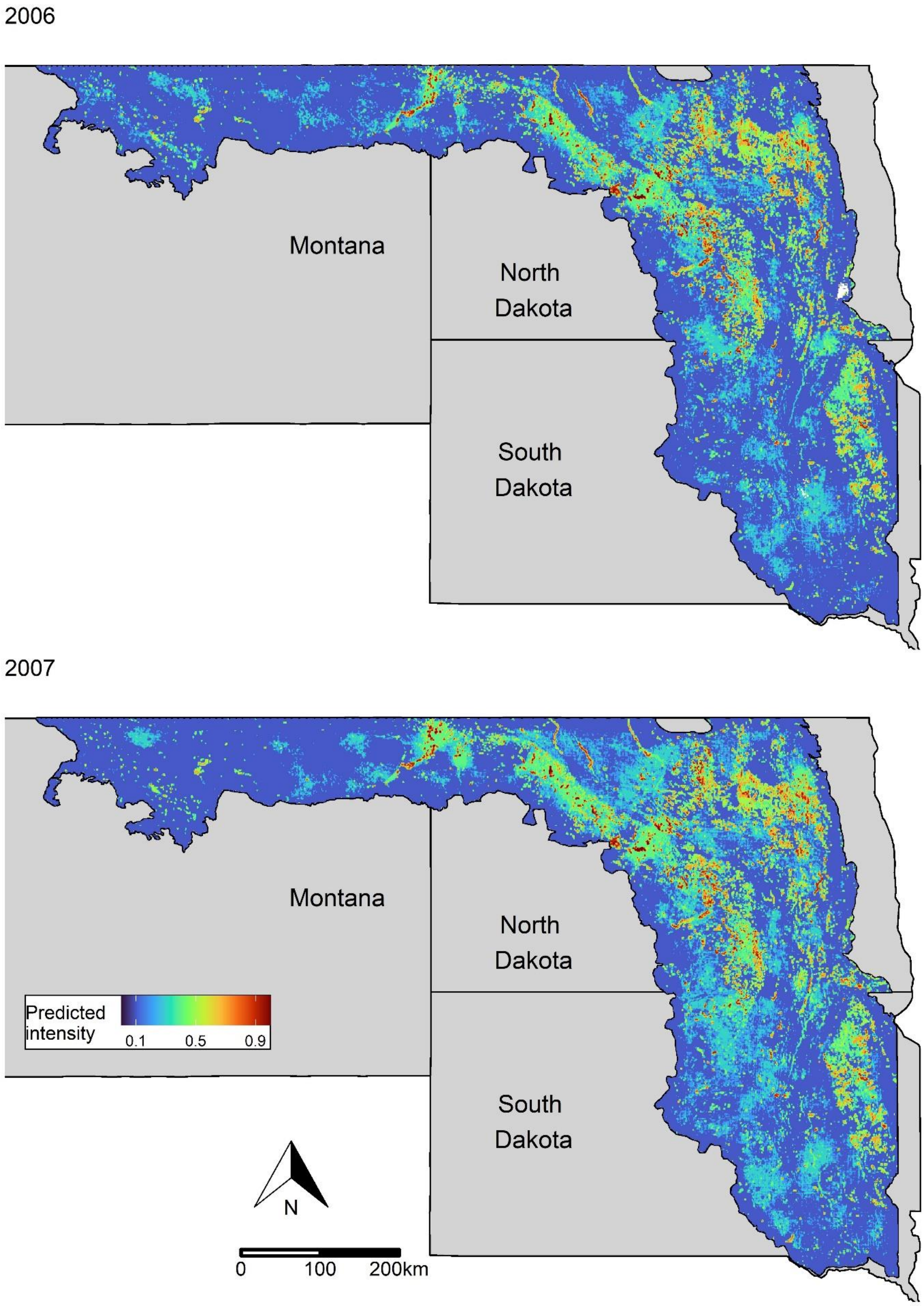

2006
Montana
North Dakota
South Dakota
2007
Montana
North Dakota
South Dakota
Predicted intensity
0.1
0.5
0.9
N
0
100
200km

2008

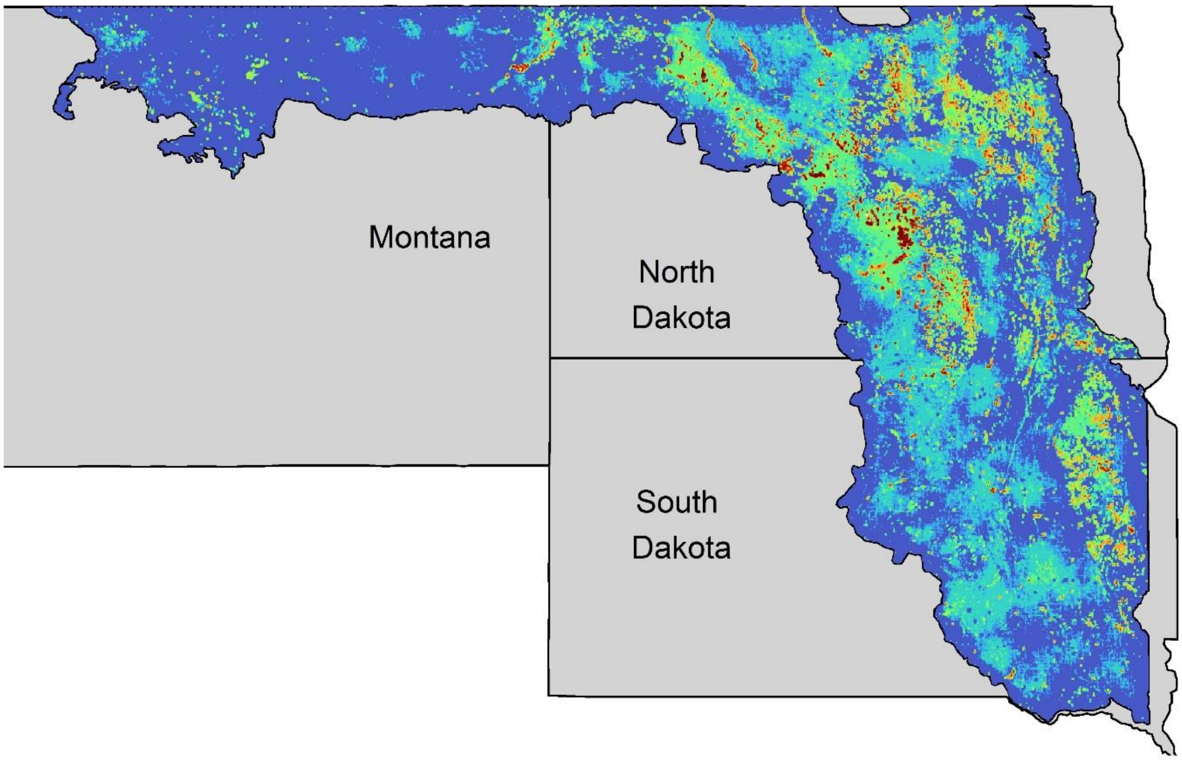
Montana
North
Dakota
South
Dakota

2009

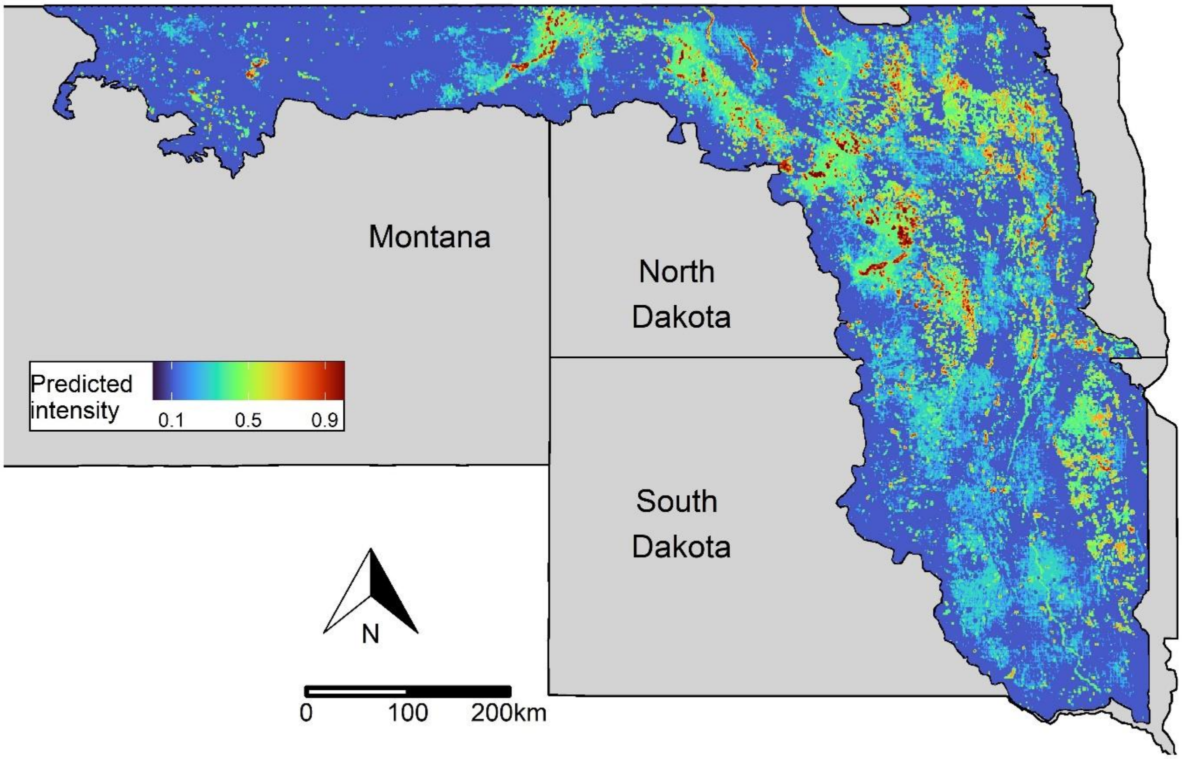
Montana
North
Dakota
South
Dakota
Predicted
intensity
0.1
0.5
0.9
N
0
100
200km

2010

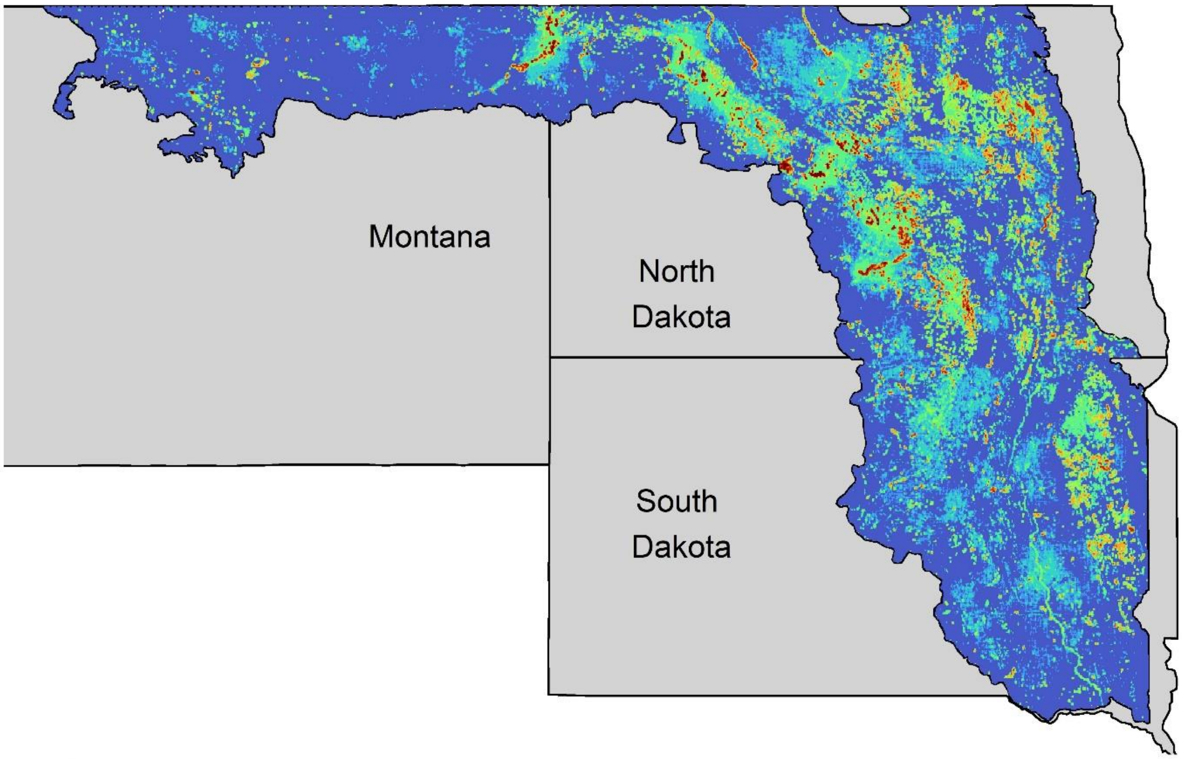

Montana
North
Dakota
South
Dakota

2011

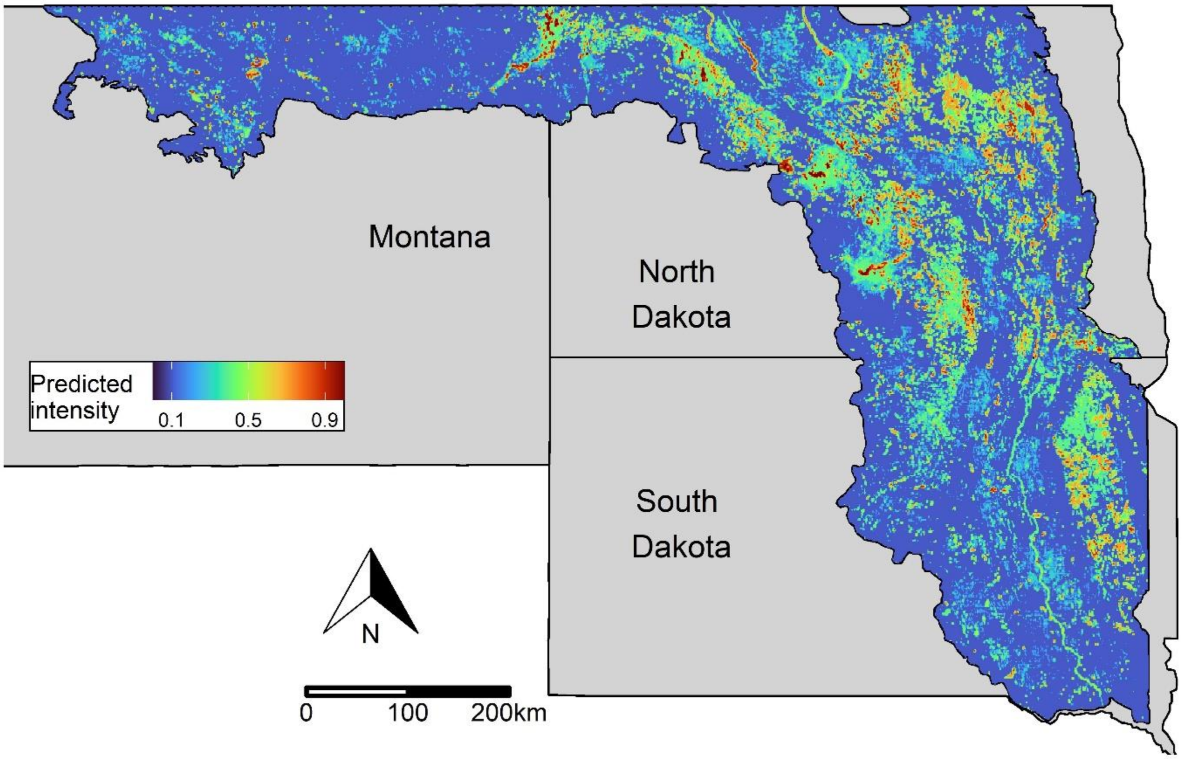

Montana
North
Dakota
South
Dakota
Predicted intensity
0.1
0.5
0.9
N
0
100
200km

2012

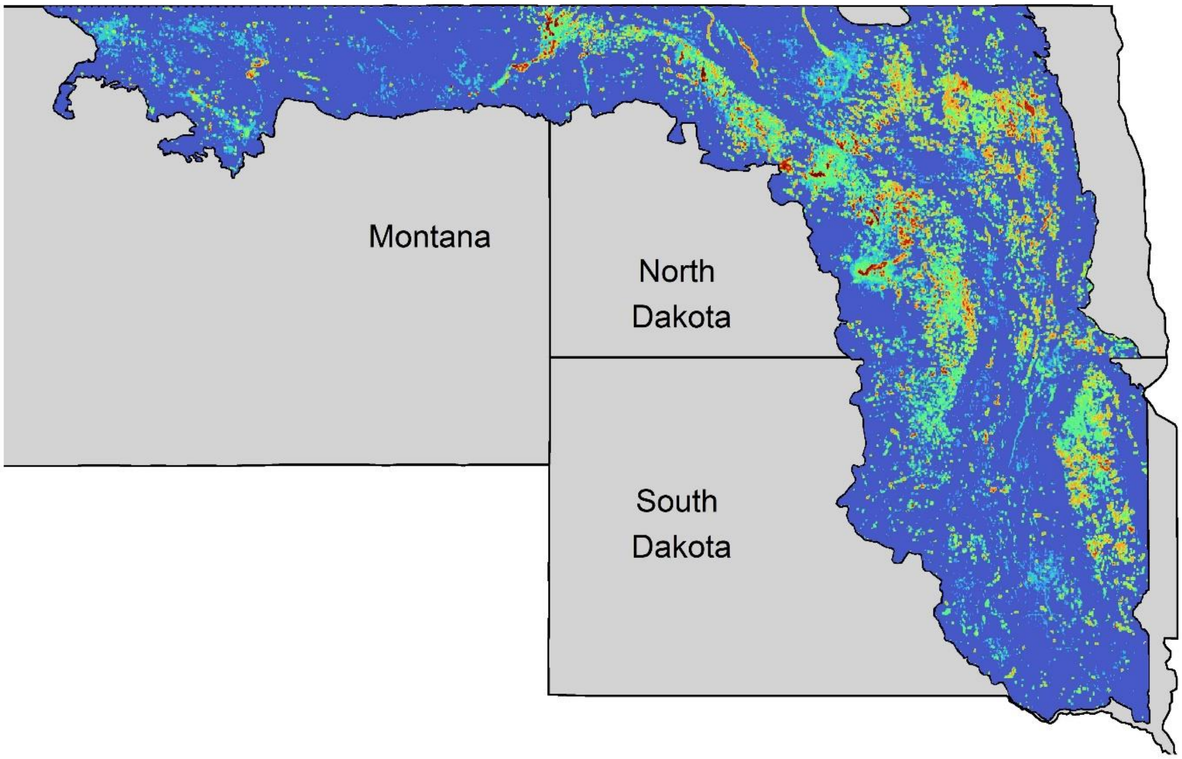
Montana
North Dakota
South Dakota

2013

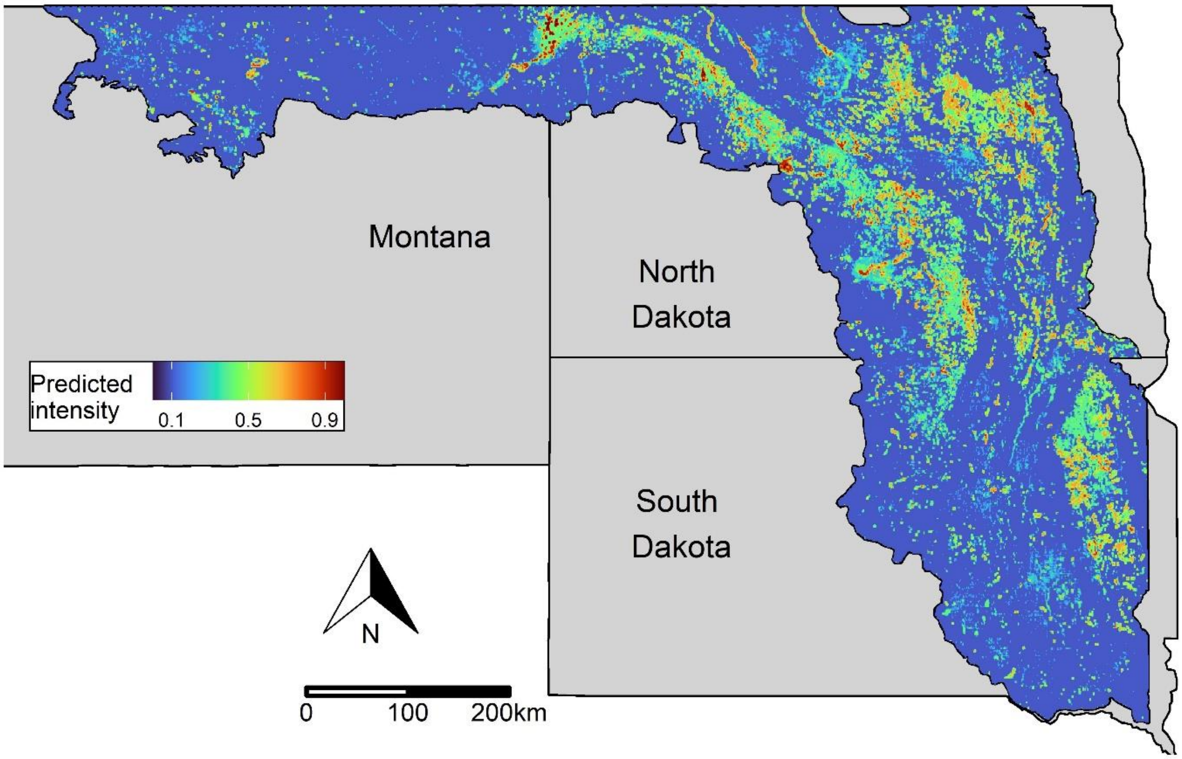
Montana
North Dakota
South Dakota
Predicted intensity
0.1
0.5
0.9
N
0
100
200km

2014

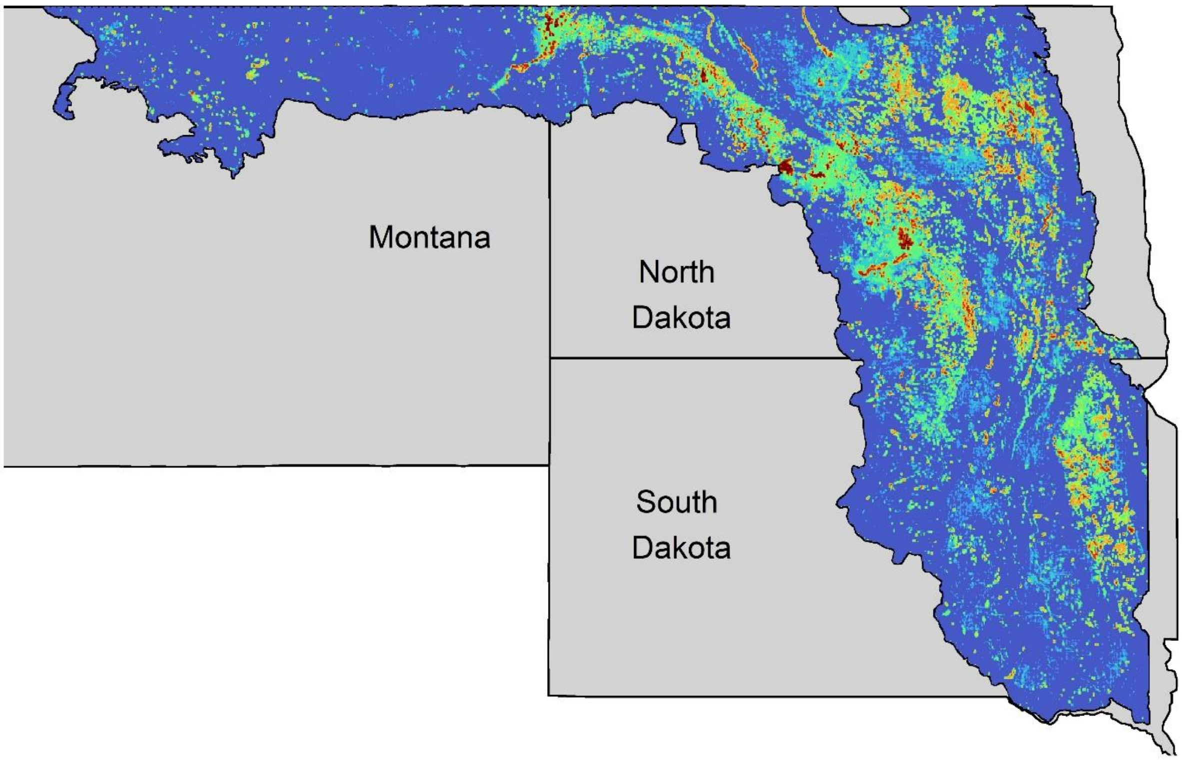
Montana
North Dakota
South Dakota

2015

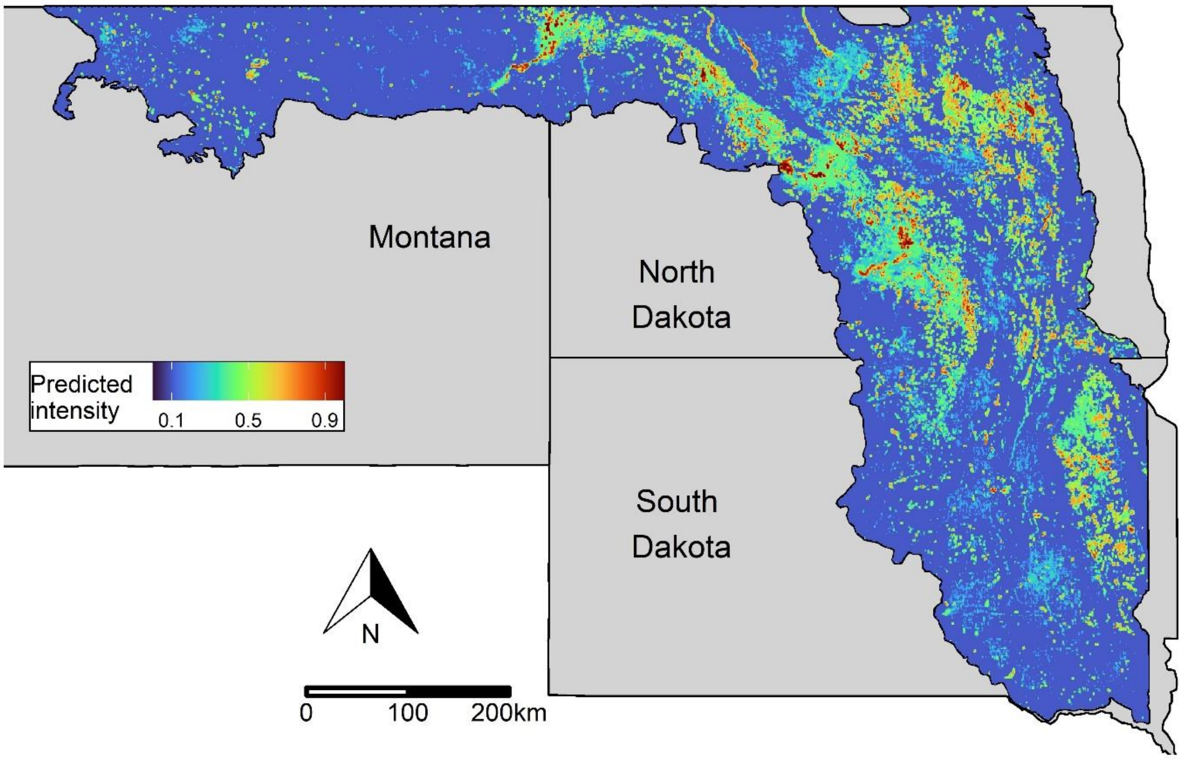
Montana
North Dakota
South Dakota
Predicted intensity
0.1
0.5
0.9
N
0
100
200km

2016

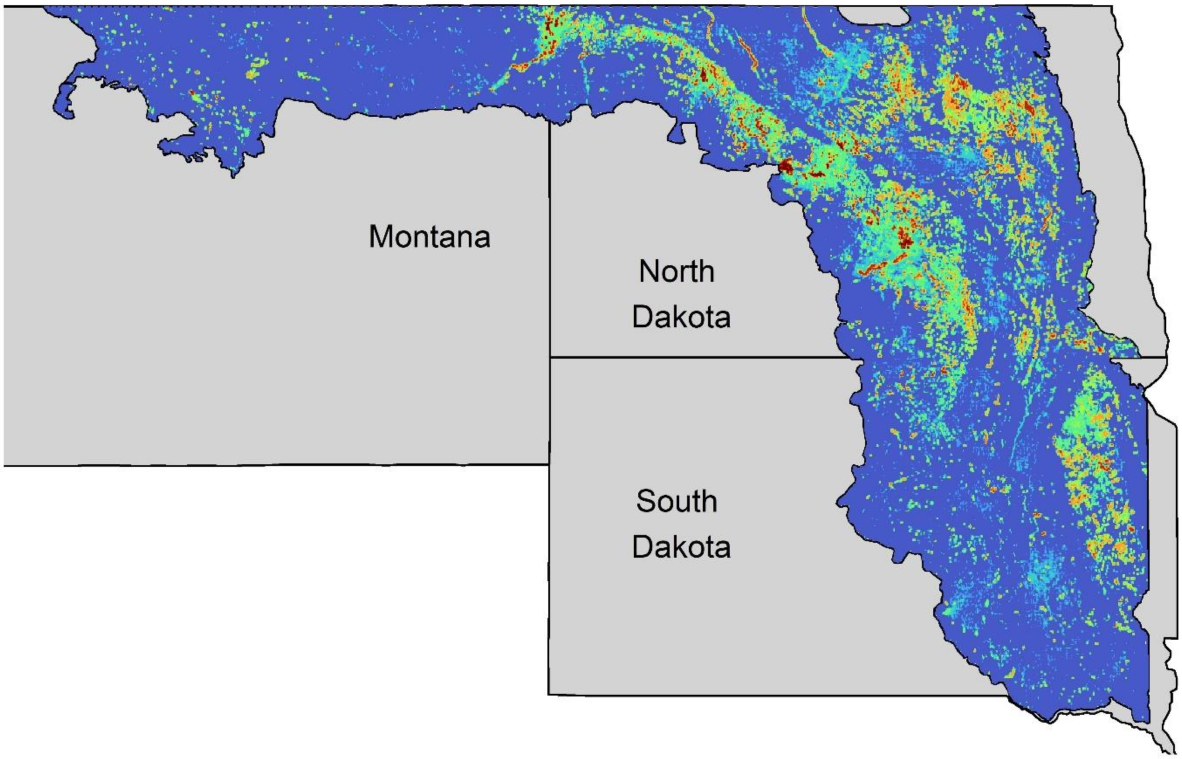
Montana
North Dakota
South Dakota

2017

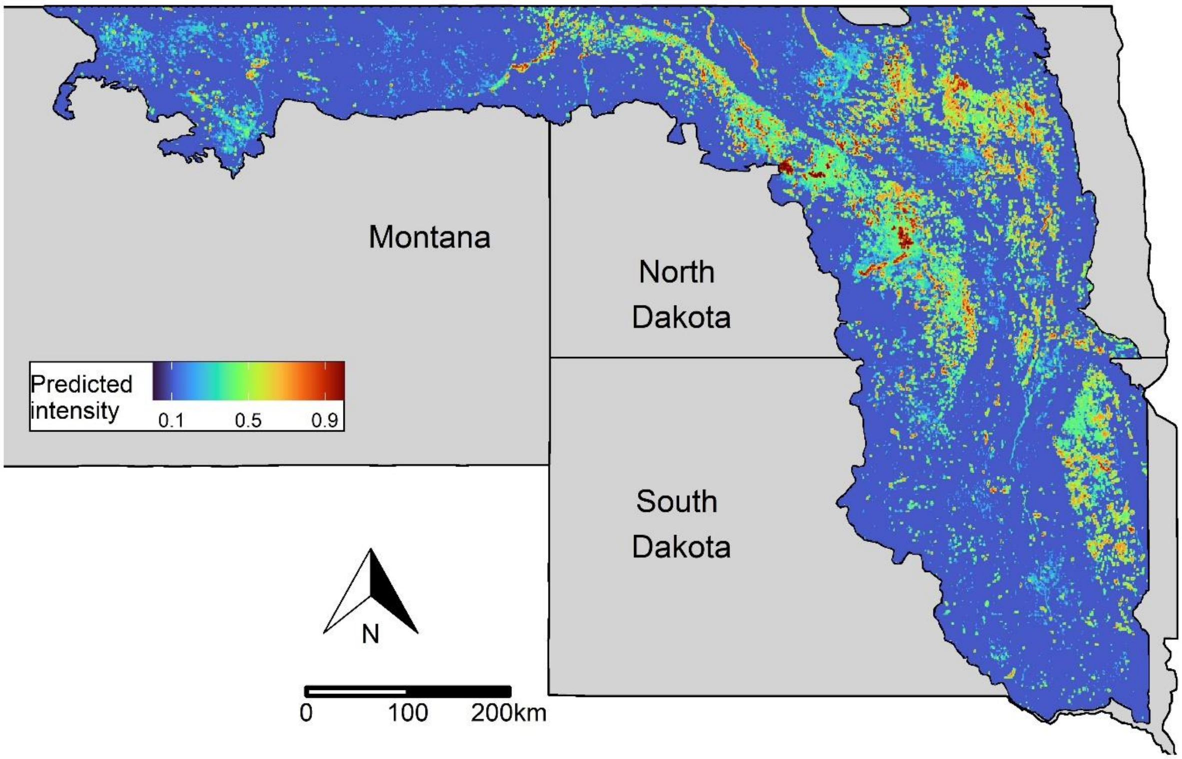
Montana
North Dakota
South Dakota
Predicted intensity
0.1
0.5
0.9
N
0
100
200km

2018

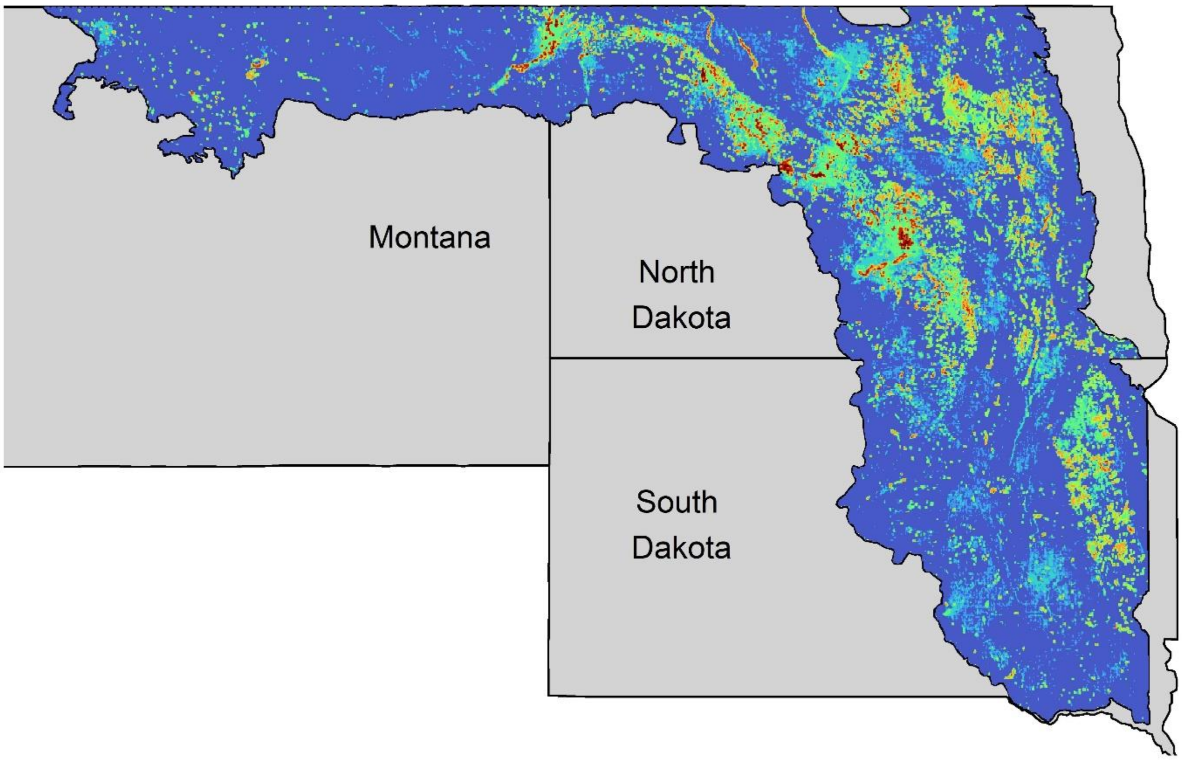
Montana
North Dakota
South Dakota

2019

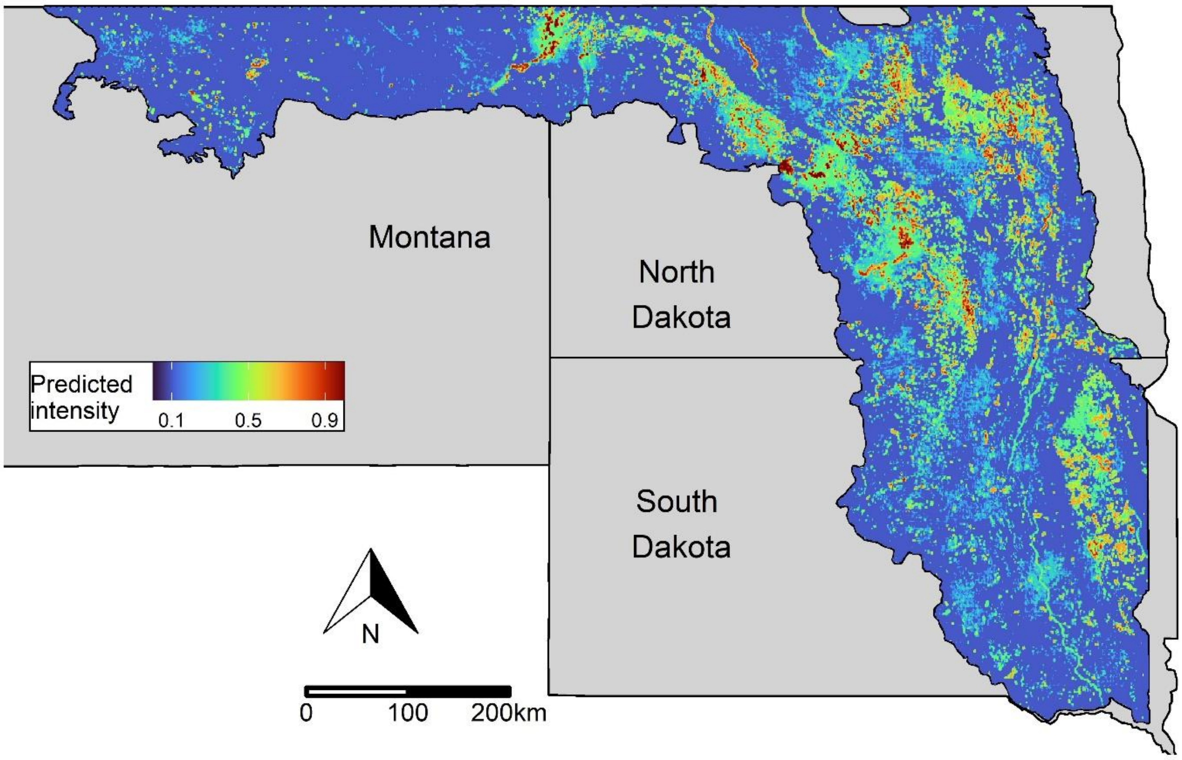
Montana
North Dakota
South Dakota
Predicted intensity
0.1
0.5
0.9
N
0
100
200km

2020

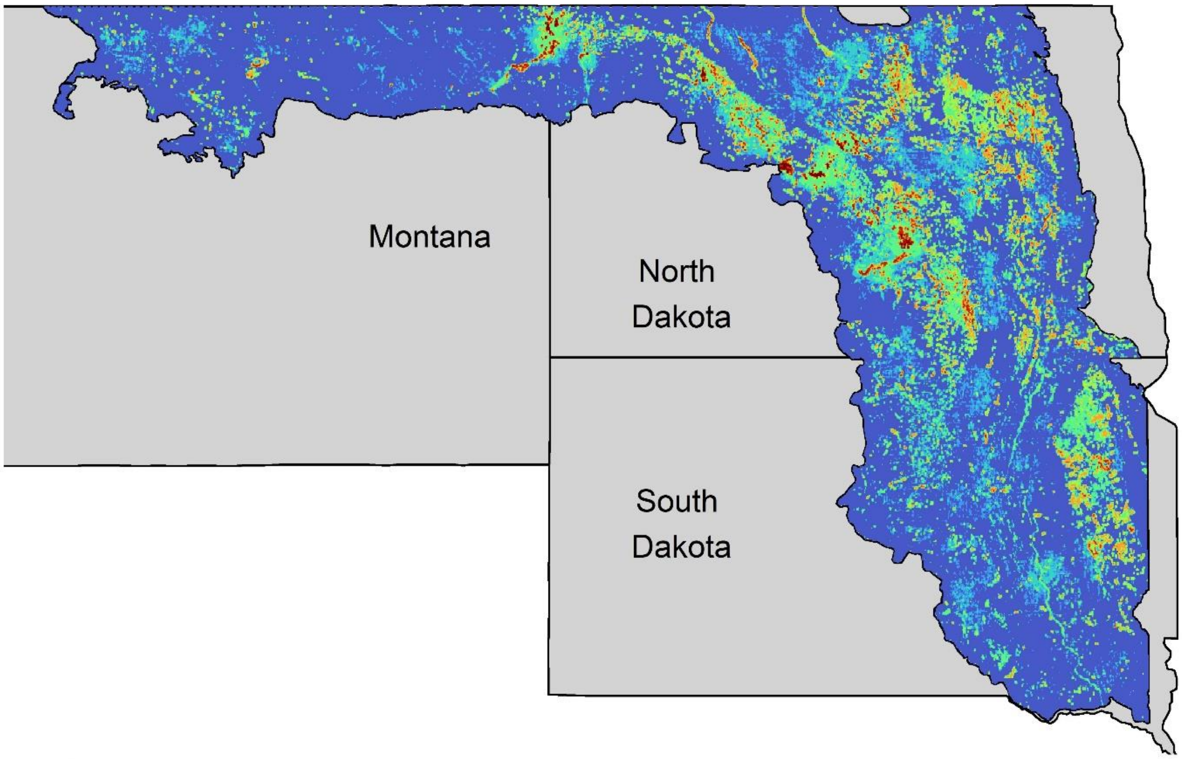

Montana
North Dakota
South Dakota

2021

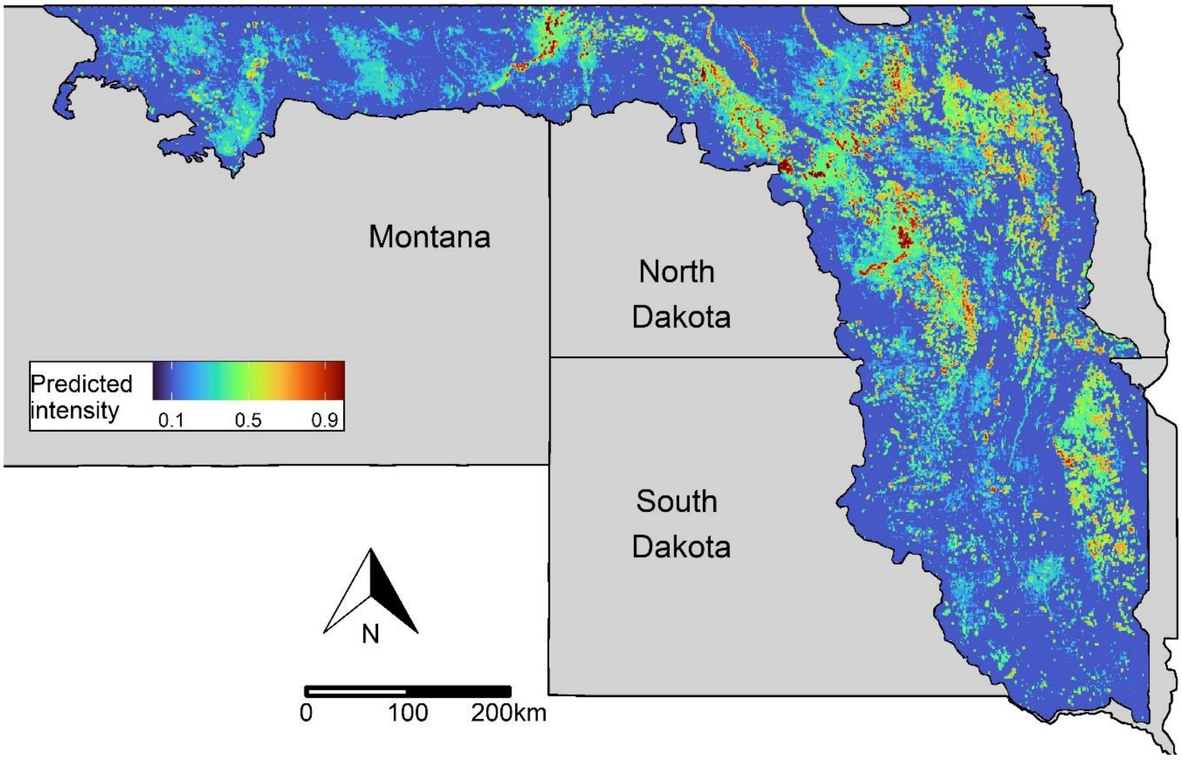

Montana
North Dakota
South Dakota
Predicted intensity
0.1
0.5
0.9
N
0
100
200km

Fig. S4. Annual spatial predictions of breeding intensity of piping plover, *Charadrius melodus*, generated from dynamic environmental explanatory layers and the temporally-varying spatial random effect. To allow for improved visualization, maps were aggregated to 900 m resolution using the mean of pixel values and normalized by rescaling values between 0 and 1. We have provided a user-friendly tool for accessing annual spatial predictions at this link: PIPL breeding habitat in the PPR (earthengine.app). Maps were generated using R (version 4.1.3; www.r-project.org).

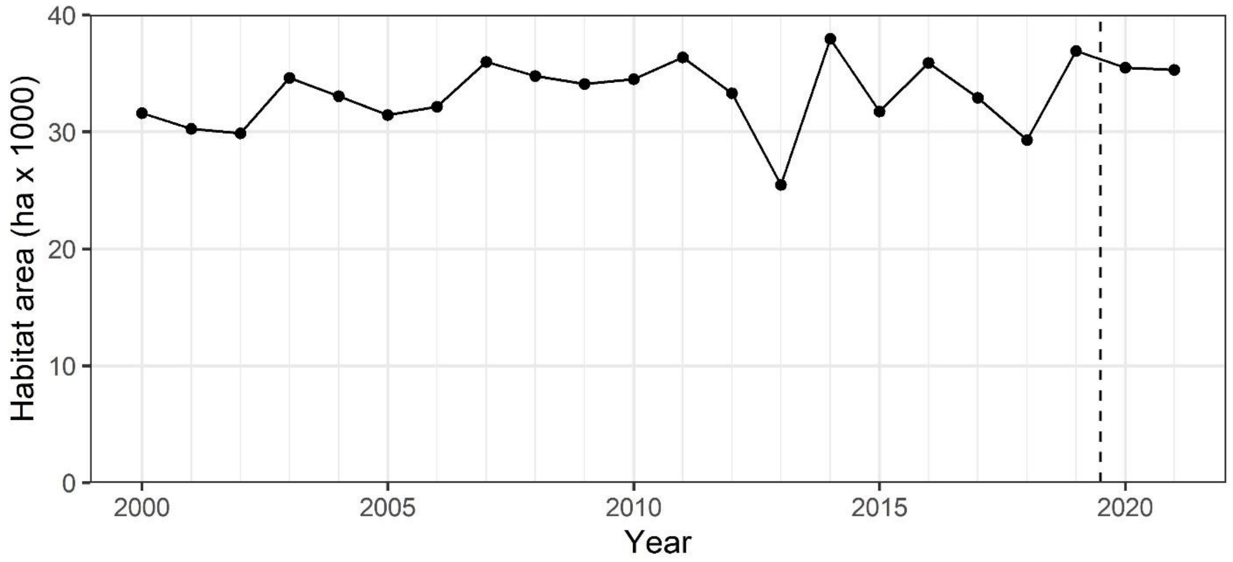

Fig. S5. Annual summaries of predicted nesting habitat area (ha x 1000) for piping plovers, *Charadrius melodus*. Estimates were calculated by converting counts of 30 m pixels greater than the habitat suitability threshold to area.

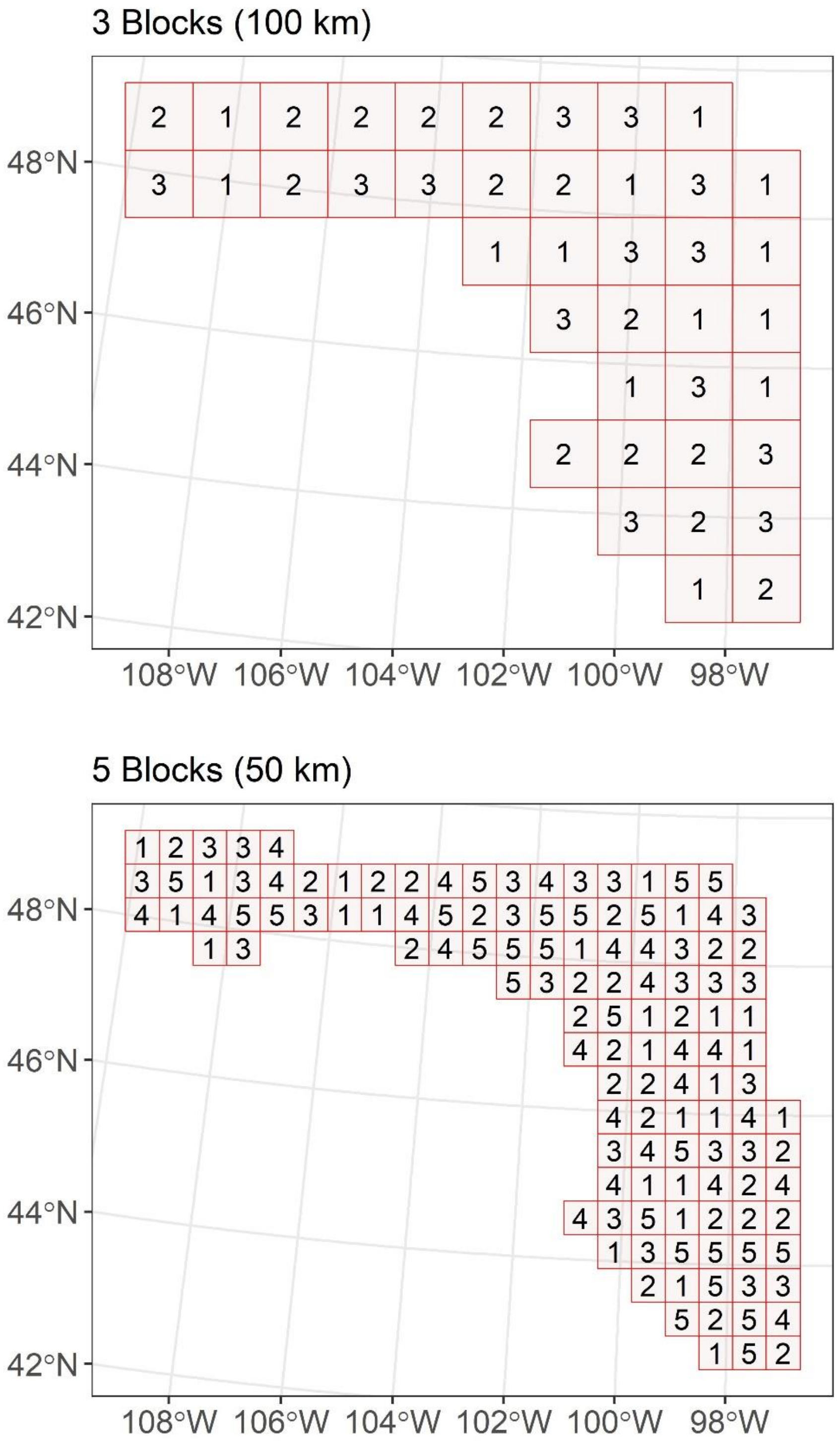

Fig. S6. Spatially separated folds used for block cross validation. Blocks were 100 km across for the 3 block procedure (top) and 50 km across for the 5 block procedure (bottom). Maps were generated using R (version 4.1.3; www.r-project.org) with the blockCV package (https://doi.org/10.1111/2041-210X.13107).

Google Earth Engine code to obtain temporally varying layers for predictor variables

Last updated: 10/18/2022

This script allows users to obtain data layers from the National Land Cover Database (NLCD, at 2–3-year intervals), Landsat, and a Digital Elevation Model (DEM). The DEM is used to derive a slope layer. Landsat imagery is used to produce annual Normalized Difference Vegetation Index (NDVI) and Dynamic Surface Water Extent (DSWE) data layers. All these layers are clipped to an area of interest (aoi).

```
// Load area of interest polygon (prediction area)--it is called "preds_area"
var ppr = preds_area.geometry();
// ---------------------------------------------------------------------
// Define AOI
// ---------------------------------------------------------------------
var aoi = ppr
// ----------------- Input user-required info ---------------------------
// Define start and end imagery acquisition dates for Landsat imagery (NDVI and DSWE) //
// Acquisition dates below are to obtain 2021 NDVI and DSWE layers
// Dates should be within Landsat TM range (Aug 22, 1982 to present)
var startDate = ee.Date('2000-01-01');
var endDate = ee.Date('2022-12-31');

// AW = acquisition window for NDVI
var AWstart = ee.Date('2021-05-01');
var AWend = ee.Date('2021-08-31');

var startDSWE = ee.Date('2021-04-01'); //
var endDSWE = ee.Date('2021-09-30'); //
// -----------------------------------------------------------------------

//////////////////////////NLCD code starts here//////////////////////////
var NLCDcollection= ee.ImageCollection('USGS/NLCD_RELEASES/2019_REL/NLCD');

// Select the landcover band
var landcover = NLCDcollection.select('landcover');
//Select the year
var listOfImages = landcover.toList(NLCDcollection.size()); //makes all NLCD years into a list
var landcover2019 = ee.Image(listOfImages.get(-1)); //select the most recent NLCD layer (last element)

print("Landcover 2019",landcover2019)
// Map.addLayer(landcover2019);

// Reclassify NLCD values - we are interested in crop and hay pasture (all other categories = 0)
// A list of pixel values to replace.
```

```
var fromList = [11, 12, 21, 22, 23, 24, 31, 41, 42, 43, 52, 71, 81, 82, 90, 95];
// A corresponding list of replacement values.
var toList = [0, 0, 0, 0, 0, 0, 0, 0, 0, 0, 0, 0, 1, 1, 0, 0];

var imgRemap = landcover2019.remap({
  from: fromList,
  to: toList
  // defaultValue: 0,
  // bandName: 'Map'
});

print("Remap Landcover 2019",imgRemap)

var landcover_clip = imgRemap.clip(aoi);

//////////////////////////NDVI CODE starts here//////////////////////////

//This field contains UNIX time in milliseconds
var timeField = 'system:time_start'

// ---------------------------------------------------------------------
// Load Landsat imagery
// ---------------------------------------------------------------------
// Define Landsat surface reflectance bands
var sensor_band_dict = ee.Dictionary({
  l9 : ee.List([1,2,3,4,5,6,17]),
  l8 : ee.List([1,2,3,4,5,6,10]),
  l7 : ee.List([0,1,2,3,4,6,9]),
  l5 : ee.List([0,1,2,3,4,6,9]),
  l4 : ee.List([0,1,2,3,4,6,9])
});

// Sensor band names corresponding to selected band numbers
var bandNames = ee.List(['blue','green','red','nir','swir1','swir2','pixel_qa']);

// Landsat 4 - Data availability Aug 22, 1982 - Dec 14, 1993
var ls4 = ee.ImageCollection('LANDSAT/LT04/C01/T1_SR')
  .filterBounds(aoi.bounds())
  .select(sensor_band_dict.get('l4'), bandNames);

// Landsat 5 - Data availability Jan 1, 1984 - May 5, 2012
var ls5 = ee.ImageCollection('LANDSAT/LT05/C01/T1_SR')
  .filterBounds(aoi.bounds())
  .select(sensor_band_dict.get('l5'), bandNames);

// Landsat 7 data are only used during operational SLC and
// to fill the gap between the end of LS5 and the beginning
// of LS8 data collection prior to SLC-off
```

```
// Landsat 7 - Data availability Jan 1, 1999 - Aug 9, 2016
// SLC-off after 31 May 2003
var ls7 = ee.ImageCollection('LANDSAT/LE07/C01/T1_SR')
  .filterDate('1999-01-01', '2003-05-31')
  .filterBounds(aoi.bounds())
  .select(sensor_band_dict.get('l7'), bandNames);

// Post SLC-off; fill the LS 5 gap
// Landsat 7 - Data availability Jan 1, 1999 - Aug 9, 2016
// SLC-off after 31 May 2003
var ls7_2 = ee.ImageCollection('LANDSAT/LE07/C01/T1_SR')
  .filterDate('2012-05-05', '2014-04-11')
  .filterBounds(aoi.bounds())
  .select(sensor_band_dict.get('l7'), bandNames);

// Landsat 8 - Data availability Apr 11, 2014 - present
var ls8 = ee.ImageCollection('LANDSAT/LC08/C02/T1_L2')
  .filterBounds(aoi.bounds())
  .select(sensor_band_dict.get('l8'), bandNames);

// Landsat 9 - Data availability 2021-10-31 to present
var ls9 = ee.ImageCollection('LANDSAT/LC09/C02/T1_L2')
  .filterBounds(aoi.bounds())
  .select(sensor_band_dict.get('l9'), bandNames);

// Merge landsat collections
var l45789 = ee.ImageCollection(ls4
  .merge(ls5)
  .merge(ls7)
  .merge(ls7_2)
  .merge(ls8)
  .merge(ls9).sort('system:time_start'))
  .filterDate(startDate, endDate);

// Mask clouds, cloud shadows, and snow
function maskClouds(img) {
  var qa = img.select(['pixel_qa']);
  var clouds = qa.bitwiseAnd(8).neq(0).or // Cloud shadow (0 = clear, 1 = contamination)
           (qa.bitwiseAnd(16).neq(0)).or // Snow
           (qa.bitwiseAnd(32).neq(0)); // Cloud
  return img.addBands(clouds.rename('clouds')); // Add band of contaminated pixels
}

// Apply mask
var coll_masked = l45789.map(maskClouds);

// Function to add variables for NDVI, MNDWI, MBSRV, MBSRN, AWESH, time and a constant to imagery
```

```
var addVariables = function(image) {
  //compute time in fractional years since the epoch
  var date = ee.Date(image.get(timeField));
  var years = date.difference(ee.Date('1970-01-01'), 'year');
  //return image with added bands
  return image
  .addBands(ee.Image(years).rename('t')) // Add a time band
  .float()
  .addBands(image.normalizedDifference(['nir', 'red']).rename('NDVI')).toFloat() // Add NDVI band
};

// Create year-specific NDVI and add it as a map layer
// First, specify the year of interest
var coll = coll_masked
    .filterBounds(aoi)
    .filterDate(AWstart, AWend)
    .map(addVariables).select('NDVI');

// Grab the max pixel value in each pixel stack and clip to study area
var ndviMax = coll.reduce(ee.Reducer.max()).clip(aoi);

// NDVI viz parameters
var palette = {min: -0.3, max: 0.6, palette: ['blue', 'white', 'green'],opacity:0.4};

Map.centerObject(aoi, 6);
Map.addLayer(ndviMax, palette, 'NDVI');

////////////////////////// DEM CODE starts here//////////////////////////
// Load DEM file, SRTM used here
var dem = ee.Image('USGS/SRTMGL1_003') //Image "SRTM Digital Elevation Data 30m"

// Construct slope image from DEM
var dem = dem.clip(aoi);
var slope = ee.Terrain.slope(dem);

////////////////////////// DSWE CODE starts here//////////////////////////

// -------------------------------------------------------------------
// DSWE coding: Jessica J. Walker, Roy E. Petrakis, Christopher E. Soulard
// -------------------------------------------------------------------
// Input: User-supplied date range (within Landsat TM availability)
// Output: Single multi-band image of monthly DSWE composites as a GEE Asset
// -------------------------------------------------------------------
// DSWE categories:
// 0 - Not Water
// 1 - Water - High Confidence
// 2 - Water - Moderate Confidence
// 3 - Partial Surface Water Pixel
```

```
// 4 - Water or wetland, low confidence
// 9 - Cloud, Cloud Shadow, or Snow (Hillshaded pixels set to 9 instead of 0)
// null - Fill (no data) ** currently left masked
// ---------------------------------------------------------------------
// Notes:
// This script uses the WGS 84 projection (EPSG:4326)
// ---------------------------------------------------------------------

// Id: USGS/SRTMGL1_003
// Dates should be within Landsat TM range (Aug 22, 1982 to present)

// ---------------------------------------------------------------------
// ---------------------------------------------------------------------
// Define AOI (var geometry)
// var aoi = ee.Geometry.Rectangle([-100, 43, -96, 47]);

// ---------------------------------------------------------------------
// Load Landsat imagery
// ---------------------------------------------------------------------
// Define Landsat surface reflectance bands
var sensor_band_dict = ee.Dictionary({
                    l8 : ee.List([1,2,3,4,5,6,10]),
                    l7 : ee.List([0,1,2,3,4,6,9]),
                    l5 : ee.List([0,1,2,3,4,6,9]),
                    l4 : ee.List([0,1,2,3,4,6,9])
                    });
// Sensor band names corresponding to selected band numbers
var bandNames = ee.List(['blue','green','red','nir','swir1','swir2','pixel_qa']);
// -----------------------------------------------------
// Landsat 4 - Data availability Aug 22, 1982 - Dec 14, 1993
var ls4 = ee.ImageCollection('LANDSAT/LT04/C01/T1_SR')
            .filterBounds(aoi)
            .select(sensor_band_dict.get('l4'), bandNames);

// -----------------------------------------------------
// Landsat 5 - Data availability Jan 1, 1984 - May 5, 2012
var ls5 = ee.ImageCollection('LANDSAT/LT05/C01/T1_SR')
            .filterBounds(aoi)
            .select(sensor_band_dict.get('l5'), bandNames);
// Landsat 7 data are only used during operational SLC and
// to fill the gap between the end of LS5 and the beginning
// of LS8 data collection
// Prior to SLC-off
// -----------------------------------------------------
// Landsat 7 - Data availability Jan 1, 1999 - Aug 9, 2016
// SLC-off after 31 May 2003
var ls7 = ee.ImageCollection('LANDSAT/LE07/C01/T1_SR')
            .filterDate('1999-01-01', '2003-05-31')
```

```
            .filterBounds(aoi)
            .select(sensor_band_dict.get('l7'), bandNames);
// Post SLC-off; fill the LS 5 gap
// -------------------------------------------------------
// Landsat 7 - Data availability Jan 1, 1999 - Aug 9, 2016
// SLC-off after 31 May 2003
var ls7_2 = ee.ImageCollection('LANDSAT/LE07/C01/T1_SR')
            .filterDate('2012-05-05', '2021-04-01')
            .filterBounds(aoi)
            .select(sensor_band_dict.get('l7'), bandNames);
// --------------------------------------------------------
// Landsat 8 - Data availability Apr 11, 2014 - present
var ls8 = ee.ImageCollection('LANDSAT/LC08/C01/T1_SR')
            .filterBounds(aoi)
            .select(sensor_band_dict.get('l8'), bandNames);

var ls_merged = ee.ImageCollection(ls4
            .merge(ls5)
            .merge(ls7)
            .merge(ls7_2)
            .merge(ls8).sort('system:time_start'))
            .filterDate(startDSWE, endDSWE);

// ---------------------------------------------------------------------
// Mask clouds, cloud shadows, and snow
// ---------------------------------------------------------------------
// https://landsat.usgs.gov/sites/default/files/documents/ledaps_product_guide.pdf
function maskClouds(img) {
  var qa = img.select(['pixel_qa']);
  var clouds = qa.bitwiseAnd(8).neq(0).or // Cloud shadow (0 = clear, 1 = contamination)
            (qa.bitwiseAnd(16).neq(0)).or // Snow
            (qa.bitwiseAnd(32).neq(0)); // Cloud
  return img.addBands(clouds.rename('clouds')); // Add band of contaminated pixels
}
// Apply mask
var img_masked = ls_merged.map(maskClouds); //.map(function(img){return img.clip(aoi)});
// ---------------------------------------------------------------------
// Calculate hillshade mask
// ---------------------------------------------------------------------
function addHillshade(img) {
    var solar_azimuth = img.get('SOLAR_AZIMUTH_ANGLE');
    var solar_zenith = img.get('SOLAR_ZENITH_ANGLE'); // solar altitude = 90-zenith
    var solar_altitude = ee.Number(90).subtract(ee.Number(solar_zenith));
   return img.addBands(ee.Terrain.hillshade(dem, solar_azimuth, solar_altitude).rename('hillshade'));
}
// Add hillshade bands
var img_hillshade = img_masked.map(addHillshade);
// ---------------------------------------------------------------------
```

```
// Calculate DSWE indices
// -------------------------------------------------------------------
function addIndices(img){
// NDVI
   img = img.addBands(img.normalizedDifference(['nir', 'red']).select([0], ['ndvi']));
// MNDWI (Modified Normalized Difference Wetness Index) = (Green - SWIR1) / (Green + SWIR1)
   img = img.addBands(img.normalizedDifference(['green', 'swir1']).select([0], ['mndwi']));
// MBSRV (Multi-band Spectral Relationship Visible) = Green + Red
   img = img.addBands(img.select('green').add(img.select('red')).select([0], ['mbsrv'])).toFloat();
// MBSRN (Multi-band Spectral Relationship Near-Infrared) = NIR + SWIR1
   img = img.addBands(img.select('nir').add(img.select('swir1')).select([0], ['mbsrn']).toFloat());
// AWEsh (Automated Water Extent Shadow) = Blue + (2.5 * Green) + (-1.5 * mbsrn) + (-0.25 * SWIR2)
   img = img.addBands(img.expression('blue + (2.5 * green) + (-1.5 * mbsrn) + (-0.25 * swir2)', {
       'blue': img.select('blue'),
       'green': img.select('green'),
       'mbsrn': img.select('mbsrn'),
       'swir2': img.select('swir2')
   }).select([0], ['awesh'])).toFloat();
   return img;
}
// Add indices
var img_indices = img_hillshade.map(addIndices);
// -------------------------------------------------------------------
// DSWE parameter testing
// -------------------------------------------------------------------
// Bitmask of 11111 = 16 + 8 + 4 + 2 + 1 = 31 = 1F in hex
// 1. ========== Function: test MNDWI ===========
// If (MNDWI > 0.124) set the ones digit (i.e., 00001)
function test_mndwi(img) {
  var mask = img.select('mndwi').gt(0.124);
  return img.addBands(mask
          .bitwiseAnd(0x1F)
          .rename('mndwi_bit'));
}
// 2. ======== Function: compare MBSRV and MBSRN ========
// If (MBSRV > MBSRN) set the tens digit (i.e., 00010)
function test_mbsrv_mbsrn(img) {
  var mask = img.select('mbsrv').gt(img.select('mbsrn'));
  return img.addBands(mask
          .bitwiseAnd(0x1F)
          .leftShift(1)  // shift left 1 space
          .rename('mbsrn_bit'));
}
// 3. ======== Function: test AWEsh ========
// If (AWEsh > 0.0) set the hundreds digit (i.e., 00100)
function test_awesh(img) {
  var mask = img.select('awesh').gt(0.0);
  return img.addBands(mask
```

```
            .bitwiseAnd(0x1F)
            .leftShift(2)  // shift left 2 spaces
            .rename('awesh_bit'));
}
// 4. ======= Function: test PSW1 ========
// If (MNDWI > -0.44 && SWIR1 < 900 && NIR < 1500 & NDVI < 0.7) set the thousands digit (i.e., 01000)
function test_mndwi_swir1_nir(img) {
  var mask = img.select('mndwi').gt(-0.44)
            .and(img.select('swir1').lt(900))
            .and(img.select('nir').lt(1500))
            .and(img.select('ndvi').lt(0.7));
  return img.addBands(mask
           .bitwiseAnd(0x1F)
           .leftShift(3)  // shift left 3 spaces
           .rename('swir1_bit'));
}
// 5. ======= Function: test PSW2 =========
// If (MNDWI > -0.5 && SWIR1 < 3000 && SWIR2 < 1000 && NIR < 2500 && Blue < 1000) set the ten-
thousands digit (i.e., 10000)
function test_mndwi_swir2_nir(img){
  var mask = img.select('mndwi').gt(-0.5)
            .and(img.select('swir1').lt(3000))
            .and(img.select('swir2').lt(1000))
            .and(img.select('nir').lt(2500))
            .and(img.select('blue').lt(1000));
  return img.addBands(mask
            .bitwiseAnd(0x1F)
            .leftShift(4)  // shift left 4 spaces
            .rename('swir2_bit'));
}
// Add all bitwise bands to image collection
img_indices_bit = ee.ImageCollection(img_indices)
            .map(test_mndwi)
            .map(test_mbsrv_mbsrn)
            .map(test_awesh)
            .map(test_mndwi_swir1_nir)
            .map(test_mndwi_swir2_nir);
// Function: consolidate individual bit bands
function sum_bit_bands(img){
  var bands = img.select(['mndwi_bit', 'mbsrn_bit', 'awesh_bit', 'swir1_bit', 'swir2_bit']);
  var summed_bands = bands.reduce(ee.Reducer.bitwiseOr());
  return img.addBands(summed_bands.rename('summed_bit_band'));
}
// Add individual bit bands to image collection and summarize
var img_indices_bit = ee.ImageCollection(img_indices)
            .map(test_mndwi)
            .map(test_mbsrv_mbsrn)
            .map(test_awesh)
```

```
            .map(test_mndwi_swir1_nir)
            .map(test_mndwi_swir2_nir)
            .map(sum_bit_bands);
// ----------------------------------------------------------------------
// Produce DSWE layers
// ----------------------------------------------------------------------
// Convert binary code into 4 DSWE categories
var img_indices_all = img_indices_bit.map(function(img){
  var reclass = img.select('summed_bit_band').remap([0, 1, 2, 3, 4, 5, 6, 7, 8, 9,
                                    10, 11, 12, 13, 14, 15, 16, 17, 18, 19,
                                    20, 21, 22, 23, 24, 25, 26, 27, 28, 29,
                                    30, 31],

                                    [0, 0, 0, 4, 0, 4, 4, 2, 0, 4,
                                     4, 2, 4, 2, 2, 1, 4, 4, 4, 2,
                                     4, 2, 2, 1, 3, 2, 2, 1, 2, 1,
                                     1, 1]).rename('dswe');
// ID cloud-contaminated pixels
  reclass = reclass.where(img.select('clouds').eq(1), 9);
// ID shaded areas
  reclass = reclass.where(img.select('hillshade').lte(110), 9);
// ID slopes
  reclass = reclass.where(img.select('dswe').eq(4) && slope.gte(5.71).or  // 10% slope = 5.71°
            (img.select('dswe').eq(3) && slope.gte(11.31)).or          // 20% slope = 11.31°
            (img.select('dswe').eq(2) && slope.gte(16.7)).or           // 30% slope = 16.7°
            (img.select('dswe').eq(1) && slope.gte(16.7)), 0);         // 30% slope = 16.7°

  return img.addBands(reclass).select('dswe');
});

print(img_indices_all.first())

function waterOnly(img) {
  var dswe = img.select('dswe')
  var datamask = dswe.neq(9)
  return img.updateMask(datamask)
}
var img_water = img_indices_all.map(waterOnly);

var Reclassify = function(img) {
  var newImage = img.remap([0, 1, 2, 3, 4], [0, 4, 3, 2, 1], null, 'dswe');
  return newImage
}

var img_reclassified = img_water.map(Reclassify)

var dswe_ic = img_reclassified.reduce(ee.Reducer.median());
var dswe_ic_clip = dswe_ic.clip(aoi);
```

```
print(dswe_ic_clip)

var dswe_viz = {min:0, max: 4, palette: ['white', 'red', 'blue']};
// Map.addLayer(dswe_ic_clip, dswe_viz, "DSWE composite");

// Convert DSWE to the proportion of water (high confidence only) at a 90m radius
var water = dswe_ic_clip.gt(3);
var nowater = dswe_ic_clip.lte(3);

// Map.addLayer(water, {}, 'Water');
// Map.addLayer(nowater, {}, 'No water');

// Moving window
var water_prop = water.reduceNeighborhood({
  reducer: ee.Reducer.mean(),
  kernel: ee.Kernel.circle({radius: 90, units: 'meters'}), // pixels; 90m radius
});

Map.addLayer(water_prop,{min:0, max:1,palette:['white','lightblue','blue'],opacity:0.4},"Proportion
water (90 m)")
```

This software has been approved for release by the U.S. Geological Survey (USGS). Although the software has been subjected to rigorous review, the USGS reserves the right to update the software as needed pursuant to further analysis and review. No warranty, expressed or implied, is made by the USGS or the U.S. Government as to the functionality of the software and related material nor shall the fact of release constitute any such warranty. Furthermore, the software is released on condition that neither the USGS nor the U.S. Government shall be held liable for any damages resulting from its authorized or unauthorized use.
